# Large Dense Periodic Arrays of Vertically Aligned Sharp Silicon Nanocones

**DOI:** 10.1186/s11671-022-03735-y

**Published:** 2022-10-16

**Authors:** Dirk Jonker, Erwin J. W. Berenschot, Niels R. Tas, Roald M. Tiggelaar, Arie van Houselt, Han J. G. E. Gardeniers

**Affiliations:** 1 Mesoscale Chemical Systems, University of Twente, MESA+ Institute, P.O. Box 217, 7500 AE Enschede, The Netherlands; 2 NanoLab Cleanroom, University of Twente, MESA+ Institute, P.O. Box 217, 7500 AE Enschede, The Netherlands; 3 Physics of Interfaces and Nanomaterials, University of Twente, MESA+ Institute, P.O. Box 217, 7500 AE Enschede, The Netherlands

**Keywords:** Periodic silicon nanocone, Nanowires, Vertical alignment, Thermal oxidation, Ion beam etching, Self-limited oxide growth

## Abstract

Convex cylindrical silicon nanostructures, also referred to as silicon nanocones, find their value in many applications ranging from photovoltaics to nanofluidics, nanophotonics, and nanoelectronic applications. To fabricate silicon nanocones, both bottom-up and top-down methods can be used. The top-down method presented in this work relies on pre-shaping of silicon nanowires by ion beam etching followed by self-limited thermal oxidation. The combination of pre-shaping and oxidation obtains high-density, high aspect ratio, periodic, and vertically aligned sharp single-crystalline silicon nanocones at the wafer-scale. The homogeneity of the presented nanocones is unprecedented and may give rise to applications where numerical modeling and experiments are combined without assumptions about morphology of the nanocone. The silicon nanocones are organized in a square periodic lattice, with 250 nm pitch giving arrays containing 1.6 billion structures per square centimeter. The nanocone arrays were several mm^2^ in size and located centimeters apart across a 100-mm-diameter single-crystalline silicon (100) substrate. For single nanocones, tip radii of curvature < 3 nm were measured. The silicon nanocones were vertically aligned, baring a height variation of < 5 nm (< 1%) for seven adjacent nanocones, whereas the height inhomogeneity is < 80 nm (< 16%) across the full wafer scale. The height inhomogeneity can be explained by inhomogeneity present in the radii of the initial columnar polymer mask. The presented method might also be applicable to silicon micro- and nanowires derived through other top-down or bottom-up methods because of the combination of ion beam etching pre-shaping and thermal oxidation sharpening.

**Graphic abstract:**

A novel method is presented where argon ion beam etching and thermal oxidation sharpening are combined to tailor a high-density single-crystalline silicon nanowire array into a vertically aligned single-crystalline silicon nanocones array with < 3 nm apex radius of curvature tips, at the wafer scale.

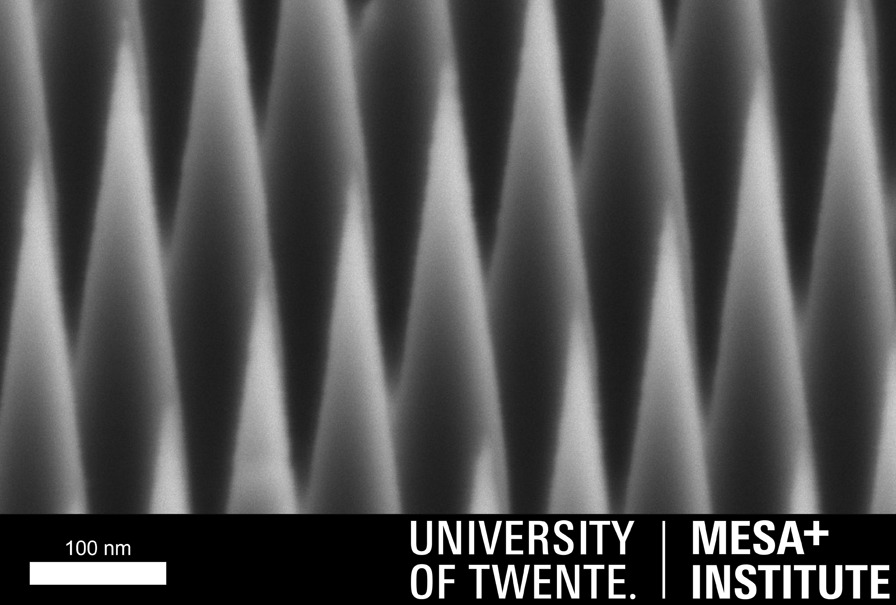

**Supplementary Information:**

The online version contains supplementary material available at 10.1186/s11671-022-03735-y.

## Introduction

Vertical silicon nanowires (SiNWs) are mostly cylindrical structures of which the diameter is in the sub-200 nm regime and the length can extend up to 50–100 times its diameter [1–3]. A limiting factor for the application of high aspect ratio (AR) wires is their decreasing flexural rigidity with increasing AR [1, 4]. If the goal is to achieve a high degree of tip sharpness while maintaining flexural rigidity, different approaches can be taken, besides reaching a large AR. Examples, where flexural rigidity and tip sharpness are important, are for instance device applications considering surface wettability. Here, contact-line pinning is largely dependent on the discontinuity of the geometrical angle presented by the substrate. In the case of high-AR structures, capillary forces might have a detrimental effect on the device performance due to the bending or deflection of the supporting wires. One way to obtain sharper nanowires is to adapt the vertical SiNW shape toward a more tapered or conical form by either a top-down or bottom-up fabrication process using anisotropic etching or epitaxial growth techniques, respectively [5–11]. These tapered silicon nanocones (SiNCs) can be used for a multitude of applications, ranging from photovoltaics to nanofluidics, nanophotonics, and nanoelectronic applications [12–17].

A popular route for fabricating high-AR SiNWs is through dry-etching techniques, where typically Bosch processes are used to achieve high etching selectivity. The characteristic of Bosch recipes is the existence of scallops on the structures’ sidewall [18]. This effect is avoided by using a continuous mode reactive ion etching (CM-RIE) recipe [11, 19]. A known problem related to CM-RIE is the presence of variation in the sidewall profile along the axis normal to the substrate. Typically etch recipes are designed toward obtaining the desired SiNW sidewall profile, by varying the process conditions during plasma etching [20–23]. For SiNC fabrication, ways of altering the sidewall profile toward a tapered structure could be done by using crystal plane selectivity in cryogenic setups [24], temporal variation of the gas mixture in a linear stepped recipe, linear stepping of other etching parameters [21, 25], or a combination of wet and dry etching utilizing corner lithography [23]. However, when optimizing an RIE recipe, the silicon (micro) loading and RIE lag are important parameters to consider and, quite often, optimized recipes do not result in similar structures under different loading conditions. Therefore, often a large amount of time is spent on recipe tuning for different device applications. For SiNWs obtained through RIE, complimentary micro- and nanofabrication techniques can be used to obtain SiNC structures. For example, part of the obtained SiNW can be converted to silicon dioxide (SiO_2_) during a thermal oxidation step, making use of the self-limiting oxidation, and sequentially stripping the oxide in an isotropic wet etch [26–31]. The pre-shape of the SiNW before the oxidation step determines the outcome of the oxidation step where, usually, the flat top of the SiNW leads to a deformation of the grown oxide due to retardation effects during oxide growth. This effect limits the maximum achievable oxide thickness. Important aspects are the initial SiNW diameter, oxidation method, and temperature, maintaining the need for RIE recipe tuning for optimizing the tip sharpness [32–35].

The complicated interplay between physical and chemical effects of ion-assisted chemical etching in RIE systems can be omitted by using inert gasses. In the past, argon has been utilized to sharpen a SiNW by using an RIE system. The authors hypothesized that the observed tip sharpening effect is due to an increased electric field at the tip, leading to an increased ion flux density [21]. In other literature works, maskless etching and sharpening of structures were suggested based on this electric field effect [36]. However, a more likely explanation for the observed tip formation and redeposition effect is the pressure range in which the inductively coupled plasma RIE (ICP RIE) functions [37]. The impeding argon ions (Ar^+^) sputter the silicon which in turn increases the pressure and decreases the mean free path length near the surface, leading to a higher lateral velocity component near the substrate that enables sputter redeposition [38, 39]. To mitigate this effect, a broad-beam gridded ion beam etching (IBE) system can be used that increases the mean free path length near the etched surface, because the plasma and substrate are in different chambers. In this way, the pressure in the plasma chamber is high enough to ignite and maintain the plasma at moderate powers, whereas the pressure in the substrate chamber is sufficiently low to achieve mean free path lengths of ~ 10^–1^ m. The ions are then directed toward the substrate by an acceleration grid, facilitating wafer-scale processing. Plasma etching systems utilizing inert gasses do not show loading dependency because the etching mechanism is purely physical [40]. However, some form of etching selectivity is found between materials due to their physical properties. The selectivity is influenced by the incident ion flux, incident ion energy, and incident angle between the surface normal of the substrate and the velocity vector of the incoming ions in an IBE system [30, 41–45]. This is another reason why the separation of the plasma and substrate chamber is beneficial, as one can alter these parameters by varying the substrate incident angle, plasma conditioning, and ion flux density and energy. Furthermore, changing the ion incidence angle and continuous concentric rotation yields an IBE process that minimizes the sputter redeposition and self-shadowing effects [30].

Regardless of the selected fabrication route, top-down or bottom-up, the pattern definition step determines the homogeneity in the structural dimensions of an array (of nanostructures), being a periodic spatial organization and the pitch between the structures. By using a maskless approach, sharp structures might be achieved but any periodic spatial organization does not seem easily possible [16, 38, 46]. From this perspective, an ultimate fabrication route toward SiNC arrays provides control over the periodicity, dimensions, sharpness as well as vertical alignment, while being able to achieve such structures on a massively parallel scale with high homogeneity. Here, vertical alignment is defined as the situation in which all apices of the structures are in the same plane, parallel to the flat (i.e. horizontal) substrate surface [47].

Because of recent developments in the field of photolithographic patterning, high homogeneity of wafer-scale periodic nanopatterns can be achieved through displacement Talbot lithography (DTL) [48–50]. Additionally, pattern transfer into an anti-reflective coating utilizing a nitrogen plasma largely simplifies the ability to achieve a highly homogeneous polymer soft mask [51]. Moreover, DTL-based mask patterns are suitable for use in CM-ICP RIE systems to achieve SiNW arrays at the wafer scale, which can then be allocated to a specific location on the substrate in areas of μm^2^–cm^2^ sizes by applying a hybrid lithography step [52, 53].

In the following, a combination of CM-ICP RIE, IBE, and thermal oxidation is used to fabricate large dense periodic arrays of vertically aligned and ultra-sharp SiNCs starting from a SiNW array. The CM-ICP RIE step is used to define a large array of periodic SiNWs, which are then tailored into a pre-shaped SiNC array by rotating, angled and timed IBE (rat-IBE), and ultimately sharpened in a thermal oxidation step. Performing the fabrication in this specific order facilitates the obtention of vertically aligned SiNC arrays. During this work, details about the rat-IBE fabrication are discussed and compared to CM-ICP RIE using argon ions. A process recipe for pre-shaping SiNCs is presented, after which thermal oxidation is conducted at different temperatures. The observations for the self-limited oxidation step suggest that a combination of the SiNC internal angle at the convex corner and the top diameter of the pre-shaped SiNC control the vertical alignment. Lastly, the obtained SiNCs are characterized by measurements of the height and tip radius of curvature. The presented SiNC array is organized in a square periodic lattice with 250 nm pitch. The silicon nanocones were vertically aligned, with mean height variations < 5 nm (< 1%) for seven adjacent nanocones, whereas the mean height variation is < 80 nm (< 16%) across the wafer scale. Apex radius of curvatures < 3 nm was measured with cone internal angles at the convex end of the SiNC equal to ~ 14°. When compared to existing nanocone configurations, similar and also smaller cone angles are reported [10, 15, 47, 54, 55]. However, the techniques are maskless and the structures non-periodic. In the case where a mask is used to obtain a periodic structure, the nanostructure surface density is an order of magnitude smaller, there is no vertical alignment, and the tip radius of curvature is not reported [12]. Considering the work cited in this article, no thorough investigation of the wafer-scale homogeneity was reported like done in this work. We conclude that the combination of the high-density, vertically aligned, ultra-sharp, high-AR, periodic SiNC arrays at the wafer-scale presented in this work is regarded as unprecedented [6, 10, 12, 14, 15, 46, 47, 54, 56–59]. The presented method, using IBE in combination with thermal oxidation, should be transferable to top-down and bottom-up derived SiNW arrays.

## Materials and Methods

### Fabrication

#### Resist Patterning

100-mm-diameter silicon (001)-oriented substrates were patterned according to the additive hybrid lithography process described in Jonker et al. [52] This comprises a patterning step by orthogonal displacement Talbot lithography (oDTL) (PhableR 100, EULITHA) and RIE of the BARC in an N_2_ plasma (TEtske, Home-built MESA+/TCO) followed by conventional I-line photolithography of a positive tone photoresist (OiR 907, Fujifilm) on a mask aligner (EVG 620, EVG).

#### CM-ICP RIE of Silicon

CM-ICP RIE of the hybrid patterned silicon substrates was performed in an inductively coupled plasma etcher (PlasmaPro 100 Estrelas, Oxford instruments). Etching and passivation were based on fluor chemistry in a mixed-mode sulfur hexafluoride + perfluorocyclobutane (SF_6_ + C_4_F_8_) process. Before etching the hybrid patterned substrate, the Plasma Pro system was cleaned with an oxygen (O_2_) cleaning program. The cleaning program ran for 15 min to minimize the influence of etching history. Subsequently, a run on a dummy wafer was performed using process conditions for 5 min to condition the process chamber. Simultaneously, the hybrid patterned substrates were treated by submerging in an aqueous 1% hydrofluoric acid (HF) solution for 30 s, to remove the native oxide, quick-dump rinsed (QDR), spin-dried and afterward immediately transferred into the Plasma Pro system and processed to prevent etching inhomogeneities in the early stage. Details of the cleaning and process conditions of CM-ICP RIE are listed in Table 1.

**Table 1:** List of settings used on the Plasma Pro system to condition the cleaning and perform the etching steps

Step	ICP power (W)	CCP power (W)	Pressure (mTorr)	SF_6_ flow (sccm)	C_4_F_8_ flow (sccm)	O_2_ flow (sccm)	Time (mm:ss)	Helium backside cooling pressure (Torr)
*Cleaning condition*								
Cleaning	1200	20	22	0	0	100	15:00	0
*Process conditions*								
Load	0	0	10^–4^	0	0	0	03:00	0
Gas check	0	0	18	9.7	20.3	0	00:10	10
Ignition	1200	30	18	19.4	40.6	0	00:02	10
Process	800	41	18	23	48	0	03:00	10
Pump down	0	0	10^–4^	0	0	0	00:30	0
Unload	0	0	10^–4^	0	0	0	03:00	0

#### IBE of Silicon Nanowires

Before a substrate was moved to the broad-beam gridded ion beam etcher (Ionfab 300, Oxford Instruments), the remainder of the hybrid mask was stripped in an oxygen plasma (Tepla 300, PVA) for 1 h at 800 W and additionally dipped in a 1% aqueous HF solution for 45 s, QDR and spin-dried. The rat-IBE was performed at the wafer-scale before thermal oxidation or on single sample SiNC arrays (1.5 × 4 mm), obtained through dicing, for characterization of the etch rate. The potential of the acceleration grid was set a 200 V_DC_, while the substrate was kept at 203 V_DC_. The rat-IBE was carried out at a process pressure of 0.17 mTorr and 300 W power was supplied to the RF generator. The measured current through the sample was ~ 3.0 mA for all experiments. During etching, the substrate was rotated at 5 rpm, and the inclination angle between the surface normal of the substrate and the surface normal of the acceleration grid was set at 45° when fabricating substrates for dry thermal oxidation or a varying angle when characterizing the etch rate on single samples.

#### Thermal Oxidation of Pre-Shaped Silicon Nanowires

Before dry thermal oxidation of the IBE-treated silicon nanowires, the substrates were cleaned with an RCA-2 cleaning, to remove ionic contaminants remnant of the various plasma treatments. The RCA-2 cleaning was performed in a volumetric (1:1:5) solution of hydrochloric acid, hydrogen peroxide, and DI water (HCl/H_2_O_2_/H_2_O) heated to 70 °C before submerging a batch of substrates. Subsequently, the substrates were rinsed by QDR and spin-dried. Organic contaminants were removed in an ozone-steam cleaning treatment. The general procedure consisted of a 30 s dip in 1% aqueous HF, QDR, 40 min of ozone-steam cleaning, a post-cleaning 30 s 1% aqueous HF dip, QDR, and finally batch spin-drying. The substrates were immediately transferred to a high-temperature atmospheric horizontal tube furnace (Omega Junior, Tempress) for dry thermal oxidation of c-Si. The substrates enter the furnace that is in equilibrium at 700 °C. The temperature was then ramped with 20 °C/min to the oxidation temperature of 850, 900, or 950 °C. After reaching the set temperature, a constant oxygen flow of 2000 sccm was applied to achieve an oxide thickness of ~ 20 nm, measured at a flat dummy substrate located in the center of the wafer boat after the substrates have left the furnace.

#### Conformal Deposition of Stoichiometric Silicon Nitride

Directly after thermal oxidation, the substrates were cleaned by ozone-steam cleaning, this time without the pre- and post-HF dip. Si_3_N_4_ was deposited in a tube furnace (TS6804, Tempress) using LPCVD at 800 °C using a 22 sccm dichlorosilane flow combined with a 66 sccm ammonia gas flow. The substrates were coated for a total time of 2 min 35 s to a thickness of 13 nm, measured at a dummy wafer located at the center of the wafer boat.

### Analyses

#### TEM Sample Preparation

Initially, the SiNC array covered with t-SiO_2_ was coated with an additional 13 nm thick layer of low-pressure chemical vapor deposited (LPCVD) stoichiometric Si_3_N_4_ and 20 nm of a sputter-deposited carbon thin film. The Si_3_N_4_ and carbon layers were added to protect the SiNCs during the vapor deposition of platinum (Pt; 2000 nm). Furthermore, the diameters of the apices of the sharpened SINWs are thin, compared to the thick layer of platinum. A thick platinum layer may scatter a large part of the electron beam and hamper the visibility of the crystalline silicon structure of the SiNCs during TEM analyses. Thus, the Si_3_N_4_ and sputter-deposited carbon film may also enhance the visibility of the apices during TEM analyses.

Before focused ion beam etching (FIB) was used to partially release an area of interest from the substrate, Pt was deposited from the vapor phase using ion beam assisted deposition, to protect the structure from secondary ion bombardments during FIB and to add structural rigidity after thinning. A wire was attached, and the TEM samples were released from the silicon substrate by SEM-assisted FIB and placed onto a TEM grid, creating a lamella. The sample was thinned to a finite size (~ 60 nm) by additional FIB. The FIB cutting was aligned at ~ 5 degrees with one of the axes of the square unit cell to increase the probability of intersecting the apex of a sharpened SiNC.

#### SEM Sample Preparation

Samples used for SEM analyses contained the t-SiO_2_ grown layer and the Si_3_N_4_ spacer layers. In case the SiNCs were observed directly, the substrates were submerged in 1% HF for 2 min, QDR, and spin-dried. The Si_3_N_4_ layers were then etched in 85% aqueous sulfuric acid (H_3_PO_4_) at 140 °C for 20 min, after which the grown t-SiO_2_ layer was etched in a 1% aqueous HF solution for 6 min. The substrate was then cleaved so that a cross-sectional image of the SiNCs could be recorded at the intersection of the cleaving plane with the device area.

## Results and Discussion

### Fabricating Silicon Nanocones

**Fig. 1:**
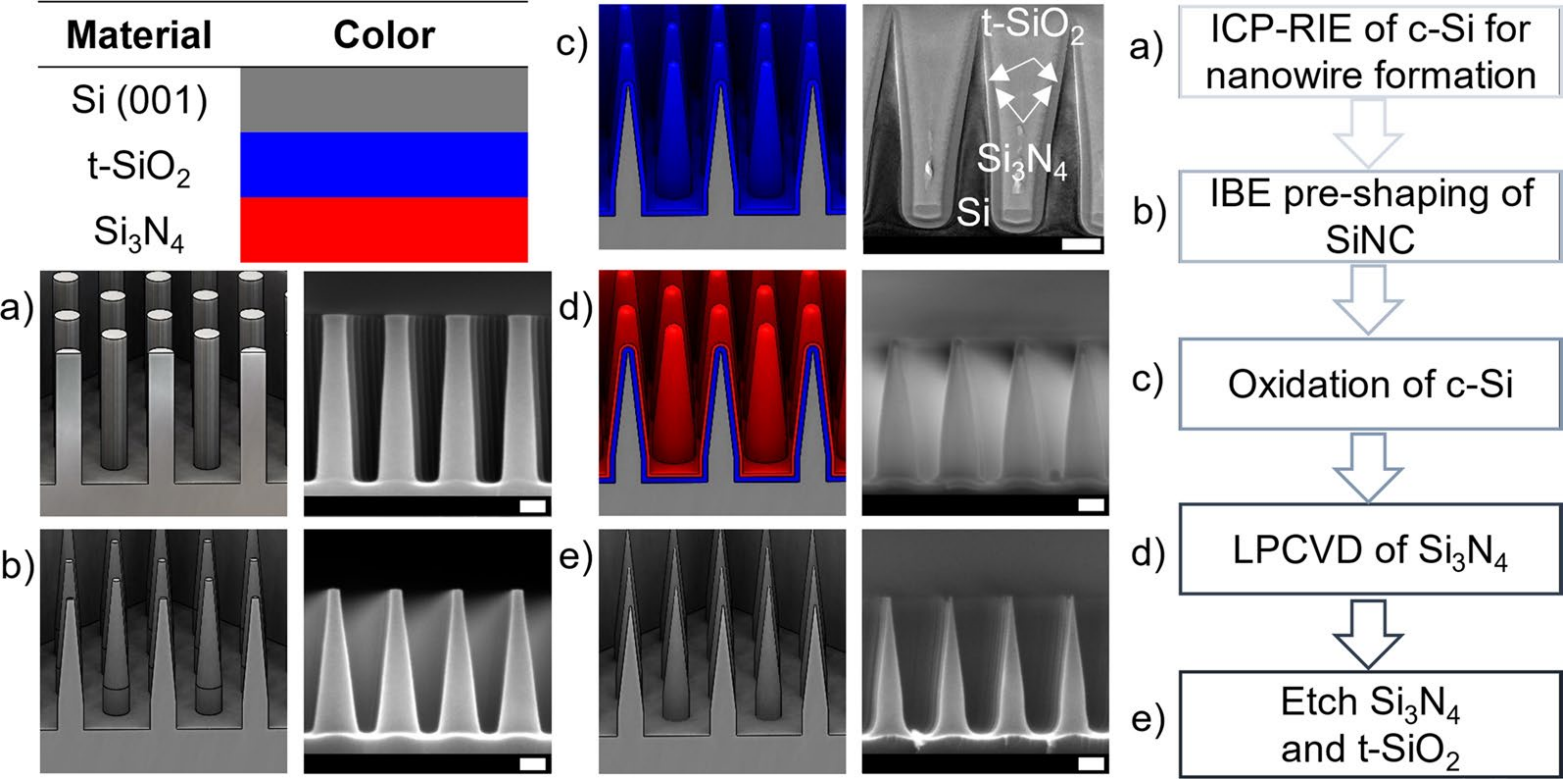
Cross-sectional schematic representation and SEM and TEM images showing the fabrication process that converts SiNWs into SiNCs. The scale bars represent 100 nm. Additionally, a flowchart is presented showing the sequential fabrication steps

The fabrication procedure entails shaping an array of single-crystalline (c-Si) SiNWs into an array of SiNCs and is visualized in Fig. 1. Starting with a pre-processed SiNWs substrate as displayed in Fig. 1(a), the consecutive steps are ion beam sharpening with Ar^+^ yielding a pre-shaped SiNC, shown in (b), thermal oxidation of silicon for further sharpening the SiNC, shown in (c), conformal deposition of stoichiometric silicon nitride (Si_3_N_4_) to protect the structures during TEM sample preparation or for future device implementation, shown in (d), and stripping of the Si_3_N_4_ and thermally grown SiO_2_ (t-SiO_2_) by wet chemical etching to finally yield the SiNC array, shown in e). Information about specific settings applied during each of the processing steps can be found in the Materials and Methods section. Observations done during the fine-tuning of the fabrication process are discussed in this results section.

The initial silicon nanowires substrate is processed according to the method described in Jonker et al*.* It consists of a 3-min CM-ICP RIE step on silicon by an inductively coupled plasma generator under an additive hybrid lithography mask. This locates the silicon nanowire arrays in several mm^2^ windows. The nanowire arrays are distributed on a 100-mm-diameter silicon substrate [52]. The result after applying this step is shown schematically in Fig. 1a). As observed in the SEM image accompanying the schematic representation, the SiNWs show extensive barreling, characterized by the curved sidewalls of the SiNWs.

### Comparison of the CM-ICP RIE in Argon with rat-IBE in Argon

**Fig. 2:**
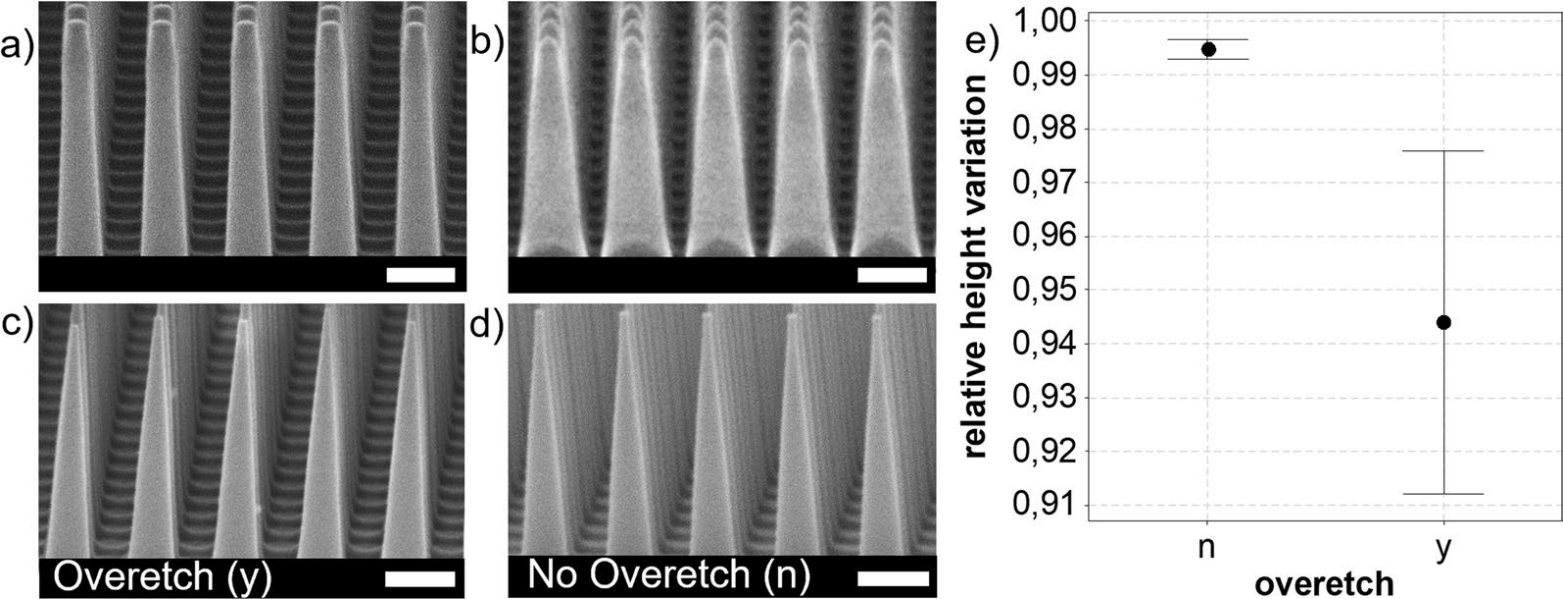
Cross-sectional images collected by SEM, after CM-ICP RIE of silicon in an argon plasma, **a**  (1 min etching) and **b** (20 min etching), and after rat-IBE of the SiNWs, **c** (overetch (y)) and** d** (overetch (n)). The scale bars represent 200 nm. The relative height variation in the case of an overetch (*y*) or no overetch (*n*) is quantified in **e** where the interval plot is based on a 95% CI with *N* = 7

To alter the sidewall to a more tapered structure, tests are conducted by applying a CM-ICP RIE Ar^+^ plasma, following the example presented by Hung et al. [21]. Some tuning of the process parameters was done to optimize performance on the used ICP RIE system (PlasmaPro 100 Estrelas, Oxford instruments). An example of the input SiNWs used for these experiments is shown in Additional file   1: Fig. SI-1 in the supporting information. The formation of a single apex on top of the individual SiNW tops was observed, together with an increase in SiNW diameter, as shown in Fig. 2a, b. Observations on the temporal behavior, shown in Additional file 1: Fig. SI-2, yield conclusions about the formation process. The apex internal angle formation process settles after some time and tip sharpness does not increase. On the other hand, the diameter of the SiNWs continues to increase, while the SiNW height decreases, leading to a decrease in the AR. This agrees with the observations by Hung et al. [21]; however, these authors hypothesize that the observed tip formation is due to an enhanced electric field effect at the edge of the SiNWs. In contrast, we follow the assumption that it is the process pressure that facilitates the sputter redeposition of the silicon. The partial pressure of sputtered silicon is highest at the center of the SiNW, which results in a lower etch rate. Studies conducted on the redeposition of metals under a polymer mask subdued to CM-ICP RIE show that indeed the redeposition process is pressure-dependent [37]. To lower the probability of redeposition, the substrates were etched in an IBE system at a much lower process chamber pressure than that during CM-ICP RIE, being 0.17 mTorr instead of 18 mTorr. The choice was made to operate the IBE at a relatively low acceleration potential of 200 V to minimize the amorphization of the crystalline silicon structure due to the impeding ions, and obtain a lower etch rate [60].

Specifications about the settings applied for the IBE step are found in the materials and methods section. Additionally, the substrate was rotated while the incident ions impede at an angle *θ*. The first experiment was to observe if any redeposition takes place. By rotating the substrate periodically around the substrate normal, the individual SiNWs are irradiated periodically from all sides, with partial shadowing by their neighboring SiNWs [30]. The substrate was etched for 10:00 (mm:ss), kept at *θ* = 45 degrees and rotated with an angular velocity of *ω* = 5 rpm. The result of this experiment is shown in Fig. 2c). The formation of an apex and positive tapering is observed, forming a pre-shaped SiNC. Compared to the structures obtained through CM-ICP RIE, no increase of diameter is observed closer to the base of the nanostructure. This observation supports the conclusion that sputter redeposition is far less probable under rat-IBE conditions. Compared to the CM-ICP RIE structures, the vertical alignment of the rat-IBE pre-shaped SiNCs is lost. This means that large height variations among neighboring pre-shaped SiNCs are prevalent. This aspect is quantified by the increased confidence interval (CI) displayed in Fig. 2e) showing the relative height variation of neighboring pre-shaped SiNCs, for the overetched case (*y*). We consider a structure to be overetched when the change in diameter is larger than the initial diameter before IBE and the top flat plateau of the SiNW is lost.

### Characterization of the rat-IBE Cycle

**Fig. 3:**
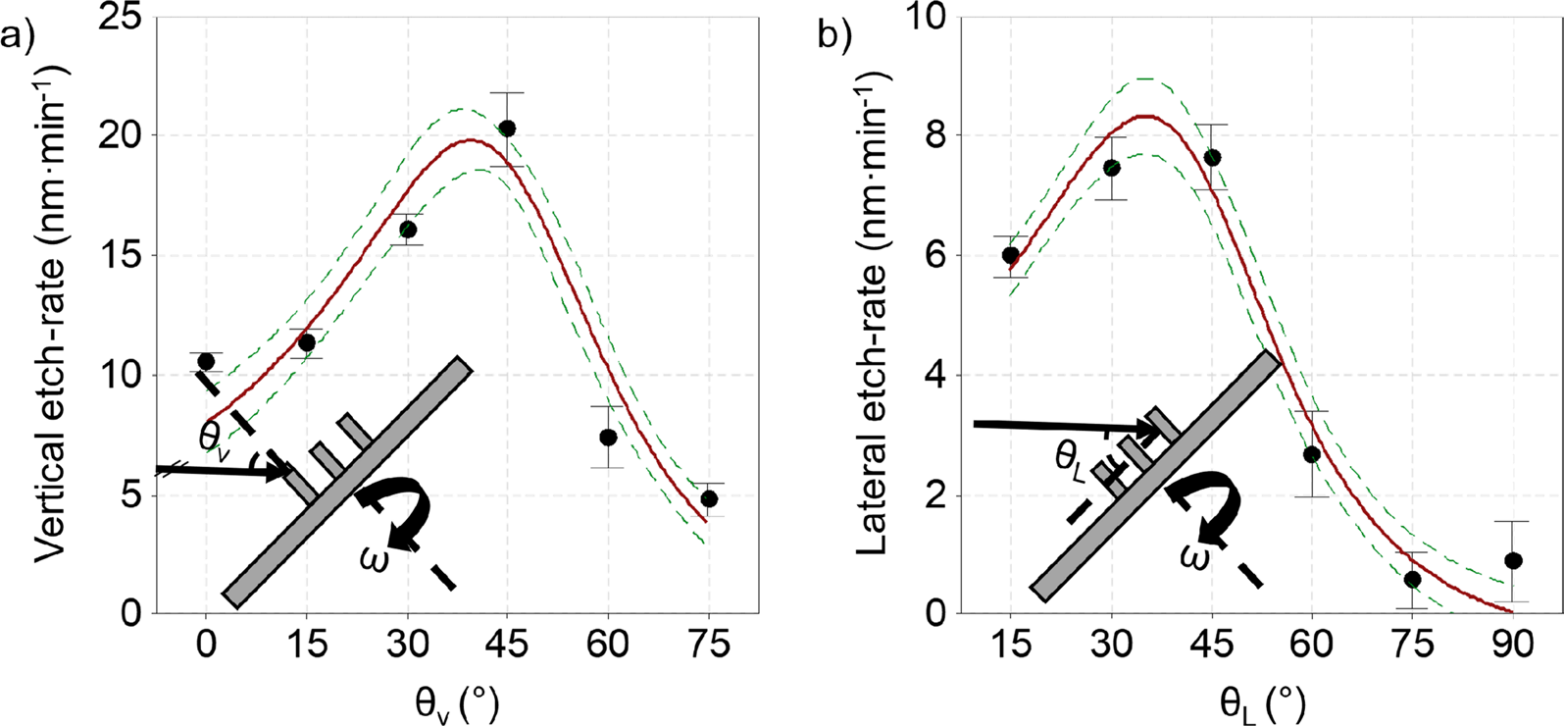
Results for the vertical and lateral etch rate as a function of the vertical (*θ*_V_) and lateral (*θ*_L_) incident angle. The interval plots represent the measured data considering a 95% CI with *N* = 6. The fitted red solid lines represent the mean etch rate. The green dotted line shows a 95% CI of the regression model

The second set of experiments focused on the structural appearance of the SiNWs after applying the IBE step for 10:00 (mm:ss) at different incident angles, *θ*. The set of input SiNWs used in these experiments is shown in Additional file 1: Fig. SI-1. For this set of experiments, the angle *θ* was decomposed into two components. An incident angle relative to the top of the SiNW, *θ*_v_, and an incident angle relative to the sidewall of the SiNW, *θ*_L_. The definitions of *θ*_v_ and *θ*_L_ are schematically represented within the insets in Fig. 3a, b, respectively. For simplicity we assume, *θ*_L_ $\equiv$ 90 − *θ*_v_ (°), and *θ*_v_ $\equiv$ *θ* (°), meaning that the angle relative to the top of the SiNW and sidewall are orthogonal and the angle relative to the top of the SiNW equals the incident angle set on the IBE apparatus.

*θ* was varied between 0° and 75° with 15° increments for which the results are shown in Additional file 1: Fig. SI-3. It was observed that with increasing *θ*, the SiNWs transfer into pre-shaped SiNCs, going from a vertically aligned flat top, in case of 0 ≤ *θ* ≤ 30°, to overetched apices for *θ* ≥ 45°. To quantify the rat-IBE etch rates, measurements of the SiNW height and top diameter, before and after applying IBE, enable the determination of the vertical and lateral etch rate. The vertical etch rate is equal to the difference in height, measured on cross-sectional SEM images, before and after IBE, divided by the total etching time. Likewise, the lateral etch rate considers the difference between the diameter of the top of the SiNW before and after IBE. To prevent the effect of overetching obscuring measurements of the top diameter for *θ* ≥ 45°, the etch time was reduced to 05:00 (mm:ss). The obtained structures and extracted dimensional parameters are shown in Additional file 1: Fig. SI-4. Note that a different set of input SiNW samples was used in these experiments visualized by the ‘no IBE’ label in Additional file 1: Fig. SI-4.

The measured angle-dependent etch rates are presented in Fig. 3a, b. For both the vertical and lateral etch rate, a maximum etch rate is found around 39 ± 7° and 35 ± 7°, respectively. The mismatch in absolute etch rates between the vertical and lateral components is assumed to be due to the periodic shadowing effect, in the case of lateral etching [30]. Three additional observations are key to deciding which *θ* is optimal. First, in Additional file 1: Fig. SI-3, an increased surface roughness at the flat silicon surface is observed for *θ* = 60°. Second, at *θ* = 30°, a gradual decrease and increase in the diameter along the vertical axis of the SiNW may facilitate vertical misalignment when combined with thermal oxidative sharpening [32, 61]. Third, an inclination appears at the edge of the SiNW for CM-ICP RIE substrates, Additional file 1: Fig. SI-2, and rat-IBE substrates etched at *θ* ≤ 30°, shown in Additional file 1: Fig. SI-3. This type of edge effect might be a trademark of the redeposition of silicon, leading to a self-shadowing effect [30]. Finally, rat-IBE was performed at *θ* = 45° to reduce the SiNW while maintaining vertical alignment. The result is shown in Fig. 2d), where the SiNW array displayed in Additional file 1: Fig. SI-1 was etched for 08:30 (mm:ss). Measurements taken before and after the timed IBE show that the vertical and lateral etch rates during this etching cycle were 20.1 and 6.1 nm min^−1^, respectively. Measurements on the relative height variation for this timed etch deliver a pre-shaped SiNC with improved vertical alignment. The relative height variations in absence of an overetch (n) are < 0.5% in a 95% CI, compared to ~ 7% when overetched (y), as shown in the interval plot in Fig. 2e). We conclude that a timed etch indeed preserves the vertical alignment and minimizes relative height variations. Moreover, the process is tunable if prior knowledge of the input SiNW structure is available. This is evident when comparing Figs. 1b–2d) where different input SiNWs have been etched.

### Oxidation of a Pre-Shaped Silicon Nanocone

The pre-shaped SiNC array is characterized by a tapered conical section with increasing diameter from top to bottom. This is similar to structures in the fabrication of scanning probe tips, where an oxidation step is key in acquiring sharp silicon tips. Single tip formation on cylindrical structures, including nanowires and nanocones, was researched extensively [34, 61–64]. From the literature, it is known that t-SiO_2_ growth on convex corners in cylindrical symmetric structures induces tensile stress in the angular direction while exerting compressive normal stress at the Si-SiO_2_ interface [33, 65, 66]. The latter limits the reaction rate at the Si-SiO_2_ interface. This effect leads to a retarded oxide growth [28, 63]. Moreover, at relatively high temperatures and extended oxidation times, the viscoelastic behavior of the oxide layer is prone to mediate stress relief. This tends to increase the growth rate [29]. In the following, a thermal oxidation step is applied to the pre-shaped SiNC. This is done to further sharpen the apices while attempting to maintain vertical alignment.

**Fig. 4:**
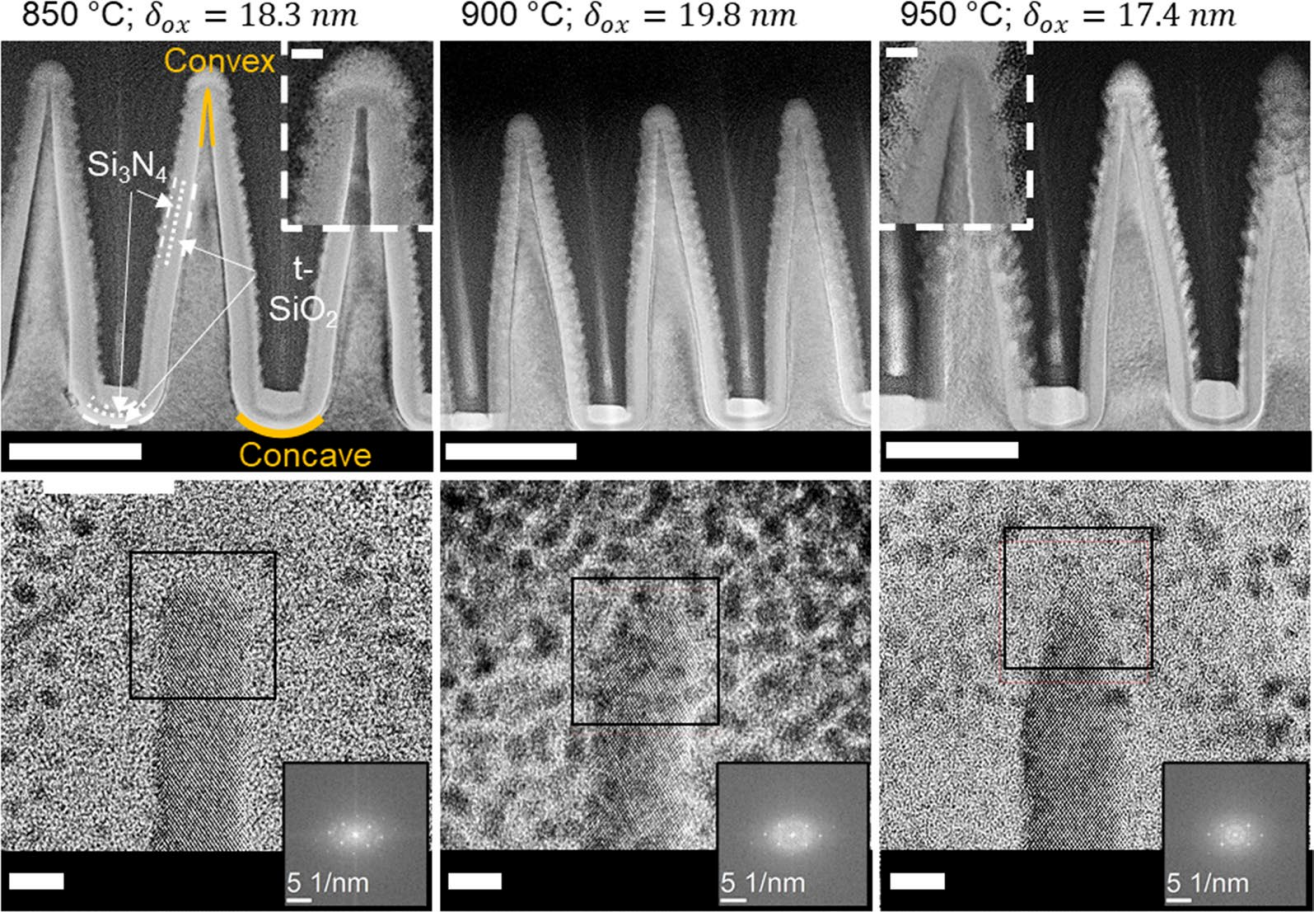
TEM images were recorded for different oxidation temperatures in the corresponding columns. The scale bars in the top-row images represent 200 nm. The insets in the top row show a magnification of the center tip with scale bars representing 20 nm. The bottom row shows a larger magnification of the same center tips as displayed in the top-row figures, whereas the scale bars represent 5 nm. The bottom-row insets display a windowed fast Fourier transform of the lattice. Analyses of the reciprocal lattice confirm that the material is crystalline silicon

The pre-shaped SiNC array used for characterizing the thermal oxidation is etched by applying a rat-IBE cycle for 10:00 (mm:ss) at *θ* = 45° to the SiNWs presented in Fig. 1a). The final top diameter of the pre-shaped SiNC array, measured from cross-sectional SEM images, was 34 ± 3 nm at the center of the substrate and is displayed in Additional file 1: Fig. SI-5. To observe the effect of the SiO_2_ growth during the sharpening of the pre-shaped SiNC, the low-temperature oxidation regime was investigated, i.e., 850 °C, 900 °C, and 950 °C. A target thickness of 20 nm is desired to fully consume the 34 nm diameter silicon column at the top of the pre-shaped SiNC. After thermal oxidation, samples are prepared for transmission electron microscopy (TEM). Ellipsometry data were recorded from (100) c-Si dummy substrates, given the thickness of the grown oxide layer, *δ*_ox_, at the flat unstructured surface for the different oxidation temperatures. The resulting TEM images together with the measured *δ*_ox_ are displayed in the top row of Fig. 4. The definition of the curvature, being convex or concave, is based on the direction of the oxygen flux during the oxidation step and is also shown in Fig. 4. Larger magnifications, as shown in the bottom row of Fig. 4, visualize the crystalline organization of the silicon. A Fourier transform of the lattice confirms the silicon crystal reciprocal organization.

Considering the results shown in Fig. 4, the oxide thickness at the convex corner is thinner than that at the concave corner, for all three investigated temperatures. This is an interesting finding, as the literature indicates that in concave corners both a restriction in mass transport occurs, because of the compressive stress in the oxide film, and a reduction in the reaction rate constant, because of compressive normal stress [34, 63, 67]. On the other hand, in convex corners, the stress in the oxide film is tensile so that solely a reduction in reaction rate due to compressive normal stress at the interface takes place. Moreover, the surface-to-volume ratio on the convex corners is larger, leading to a relatively higher concentration of oxidizing species at the convex corner. Therefore, more pronounced retardation is expected in the concave corner [63]. An example was found in the works of Zhao et al*.* The oxidation of a step edge, containing a convex and concave corner, roughly perpendicular to each other, shows a less pronounced retardation effect of the convex when compared to the concave corner [68]. In experimental work conducted by Kozhummal et al*.*, oxidation of convex corners with an internal angle of 70.6° shows already a more pronounced retarded growth at the convex corners. However, the retardation becomes less pronounced at increased temperatures [69]. In the work presented here, the retardation effect on the convex corner is even more pronounced. However, the internal angle of the convex corner of the SiNC changes from ~ 90° at the flat top of the rat-IBE pre-shaped SiNC to ~ 14° for the oxidized SiNC. This is a much smaller angle compared to that of Zhao and Kozhummal. This leads to the hypothesis that a small internal angle may show more pronounced retardation on convex corners. A more careful look at a higher magnification, like in the inset panels in the top-row and bottom-row images of Fig. 4, reveals that, at the lowest investigated temperature (i.e., 850 °C), the SiNC seems to bend outwards at the apex.

**Fig. 5:**
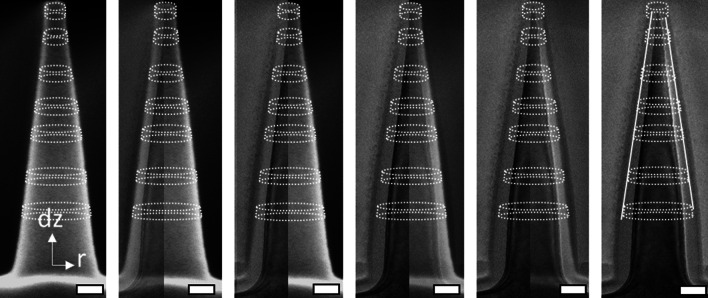
Overlapping cross-sectional SEM and TEM images, in which the transparency of the SEM image is changed from 0 to 100% for both the left-half and right-half of the image. On the left side solely the SEM image is seen, whereas on the right side solely the TEM is observed. A coordinate system is drawn together with visual aids representing sliced vertical conical sections. The scale bars represent 50 nm

Cui et al*.* found that for SiNWs with a starting diameter of 30 ± 5 nm, the dry oxidation process self-limits to a core diameter of approximately 6 nm at 850 °C. To investigate the self-limiting behavior at 850 °C, a series of thermal oxidation steps is performed at different oxidation times. From the cross-sectional SEM images presented in Additional file 1: Fig. SI-6, it is observed that the t-SiO_2_ film continues its growth at the planar silicon substrate. However, the morphology of the SiNC does not change its appearance much. In earlier works, Agache et al. showed that when the SiNC is segmented into small vertical conical sections with finite thickness dz, the thickness of the grown oxide increases with increasing initial diameter. Such an approach may lead to the accurate prediction of the oxide morphology after model calibration [64]. An example of this is shown in Fig. 5, where a series of cross-sectional SEM and TEM images were overlaid. This analysis shows a gradient oxide thickness, where the oxide is thinner wherever the SiNC diameter is smaller, in accordance with Agache*’s* model. However, Fig. 5 also reveals that the t-SiO_2_ barely expands in the outward direction, where the t-SiO_2_-ambient interface stays approximately in the same location as the initial pre-shaped c-Si-ambient interface. The growth is mainly in the inward direction, toward the c-Si-SiO_2_ interface. This is different from what is expected based on accepted models [63] where the t-SiO_2_ does expand in the outward direction.

The hypothesis is made that the final SiNC shape, in the case of the 850 °C experiments, both the initial diameter of the pre-shaped SiNC and the small internal angle of the convex corner determine the final appearance [63, 64]. For higher oxidation temperatures, the diameter of the self-limited core is smaller and achieved faster [62, 64, 70]. For the highest oxidation temperature (i.e., 950 °C) the outward bending at the tip seems absent, as observed in Fig. 4. Thus, we conclude that in such a case the self-limiting condition is not yet met.

Lastly, at the highest temperature, 950 °C, parts of the SiNC core are fully oxidized, as is displayed in Additional file 1: Fig. SI-7. It is known that the rate constants for the thermal oxidation of crystalline silicon are crystal plane dependent and thus may vary over the tapered side of the SiNC [71–73]. Whenever a fast-oxidizing plane is encountered, growth may be accelerated. However, this also increases the stress built up in the layer which lowers the reaction rate. Another effect that might be considered is the stress-induced yielding of the silicon crystal delivering a fracture and therefore a fast-oxidizing interface, as a crack will immediately relieve some of the stress accumulated in the t-SiO_2_.

### Determination of the Apex Radii of Curvature

**Fig. 6:**
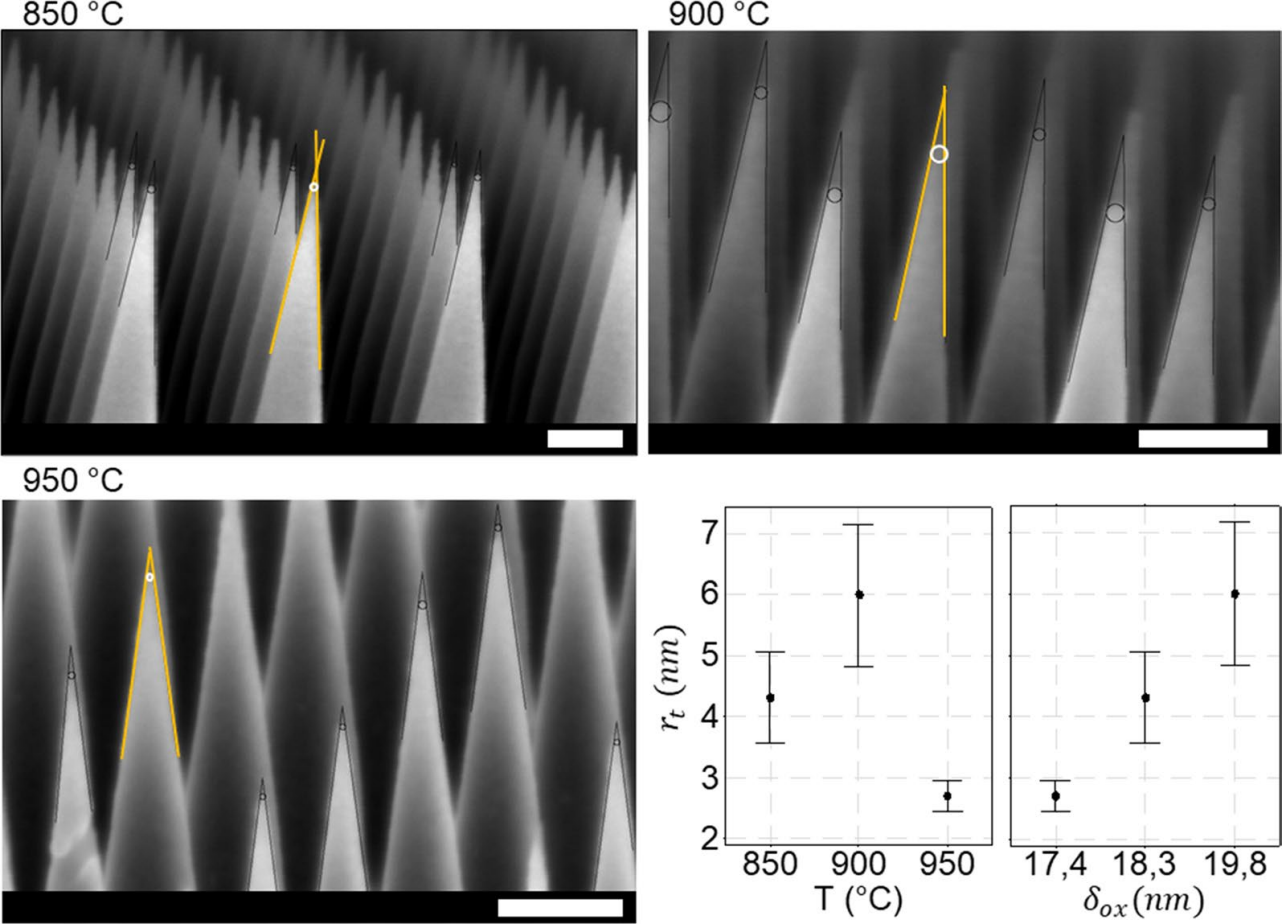
SEM images of stripped SiNC tips were recorded for the different oxidation temperatures. The graphs in the bottom right show interval plots of the measured tip radii plotted against the different oxidation temperatures versus the oxide thickness measured on the flat dummy substrate. The CI is set at 95% with *N* = 7. The scale bars represent 100 nm

To determine the apex radius of curvature, *r*_t_, high-resolution SEM images of the stripped SiNCs are recorded at the cross section of a cleaved substrate. A set of tangential lines are drawn after which a circular section is manually fitted to the apex to measure *r*_t_. The processed images are shown in Fig. 6. Additional unprocessed images are shown in Additional file 1: Fig. SI-8. The data for *r*_t_ as a function of the oxidation temperature are also shown in Fig. 6, which show that no clear trend is observed as a function of the oxidation temperature. However, different oxidation thicknesses are observed on the flat dummy substrates as shown in Fig. 4. Plotting the data versus the obtained oxidation thickness at the dummy substrates reveals that an over-oxidation of the substrate yields a blunting effect whenever the self-limiting behavior is absent. According to the SEM recorded measurements on the pre-shaped SiNCs, the top diameter was 34 ± 3 nm before oxidation shown in Additional file 1: Fig. SI-5. This value is close to twice the total thickness of the oxide layer, considering that the Si-SiO_2_ interface meets radially at the apex of the SiNC for the experiment conducted at 950 °C. For the 900 °C experiments, an additional 2.4 nm of oxide was grown from both sides, leading to an over-consumption of the silicon within the crystalline SiNC core and an uncontrolled value of *r*_t_. The choice is thus whether to time the oxidative process or set a temperature to obtain self-limited growth.

### Wafer-Scale Determination of the SiNC Height Distribution

**Fig. 7:**
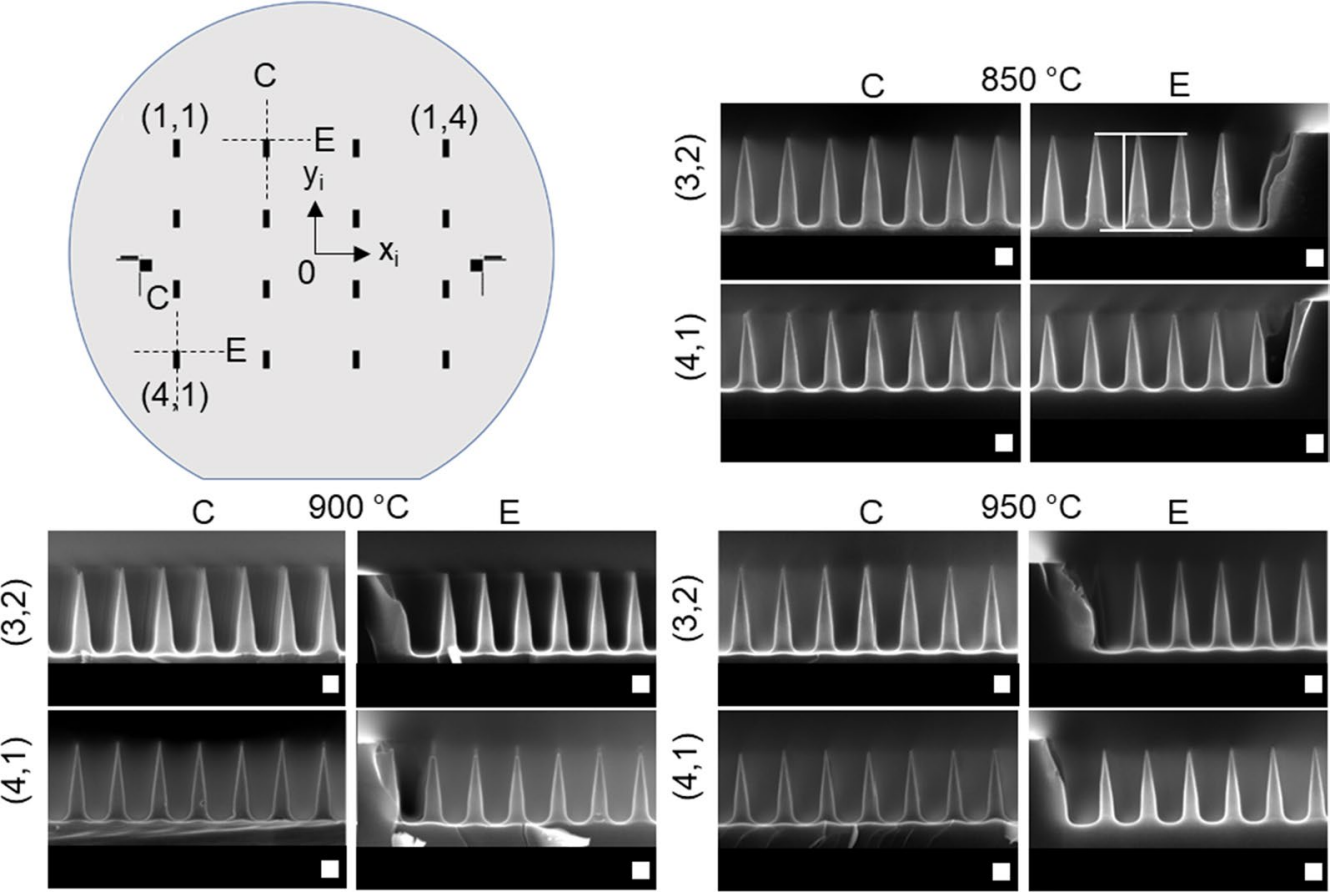
A top-view schematic representation of a 100-mm-diameter silicon substrate map is shown in the top-left corner. The black rectangular boxes indicate the SiNC arrays (size 1.5 × 4 mm^2^) on the substrate where the SiNC arrays are present. Two dotted lines show examples of the locations where the cleaving planes are supposed to intersect the nanocone arrays. Cross-sectional SEM images displayed in this figure were recorded at the cleaving planes, showing SiNC arrays fabricated at oxidation temperatures of 850, 900, and 950 °C and recorded at different locations. Height measurements of the coated and stripped SiNC arrays are conducted, as shown in (850 °C; C; (3,2)). The scale bars represent 100 nm

The wafer-scale performance of the fabrication process is characterized by fabricating SiNC arrays at different locations on a 100-mm-diameter single-crystalline silicon substrate. Using additive hybrid lithography [52], sixteen 1.5 × 4 mm^2^ etch SiNC arrays are allocated to discrete substrate positions, as shown schematically in Fig. 7. The substrates were cleaved in the 4 mm direction, intersecting the SiNC arrays. The positions are discretized by their row and column index (row, column) also schematically shown in Fig. 7. Cross-sectional SEM images were collected at the cleaving plane of the discretized SiNC arrays, close to either the edge (*E*) or the center (*C*) of the array. Together with the metadata provided by the SEM system, the location where the cross-sectional images are recorded can be derived. The metadata provides an (*x*, *y*)-SEM-stage position at which measurements are taken. Using this method, the radial coordinate, ${r}_{\mathrm{c}}$, is calculated and defined to be the radial distance from the center of the mask design, denoted with 0 in Fig. 7, by:

1 $$r_{{\text{c}}} \equiv \sqrt {x_{i}^{2} + y_{i}^{2} }$$

where ${x}_{i}$ and ${y}_{i}$ are Cartesian coordinates corresponding to the mask origin, as schematically represented in Fig. 7. The calculated uncertainty in the radial position is ± 0.8 mm. It is assumed that the mask and substrate origin are aligned in the *x*- and *y*-plane, so that *x* = *x*_i_ and *y* = *y*_i_; however, some misalignment of the origins may be present. The data presented in Additional file 1: Fig. SI-13 consider height measurements on the stripped SiNC arrays plotted against the discretized positions. It is observed that the height distribution has a parabolic shape, but with different ${r}_{\mathrm{c}}$ for the parabola maximum. The cause for this could be the misalignment of the mask origin with respect to the substrate origin.

**Fig. 8:**
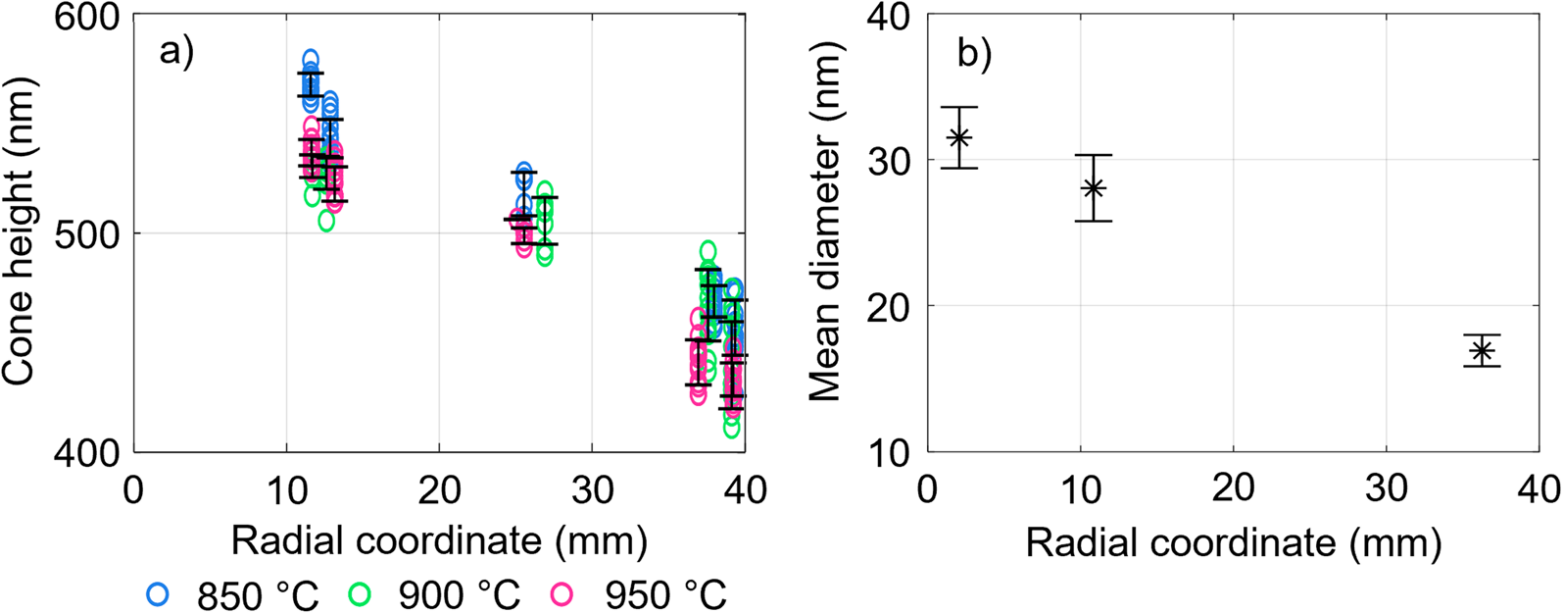
**a** Interval plot of the measurements taken on the stripped SiNC heights, for three oxidation temperatures, measured at different radial coordinates as calculated with Eq. (1). A 95% CI is given with *N* = 14 individual cones for the interval plots. **b** Interval plot of the mean diameter of the top of the pre-shaped SiNC array before oxidation. The radial coordinates are calculated as shown in Eq. (1). The diameters are measured by an automated MATLAB script. The CI is given with *N* > 50 for individual intervals

Figure 8a) shows the measured SiNC heights as a function of ${r}_{\mathrm{c}}$. For all oxidation temperatures, the SiNC height decreases with increasing radial distance from the center. Generally, the mean SiNC height decreases with increased oxidation temperatures. Also, there is a larger gradient of the height as a function of ${r}_{\mathrm{c}}$ for higher oxidation temperatures. Furthermore, the measured variance increases with increasing distance from the center of the substrate. In Fig. 8b), the initial mean diameter of the top of the IBE pre-shaped SiNC prior to thermal oxidation is plotted for different positions. This diameter is extracted using an automated MATLAB script, as shown in Additional file 1: Fig. SI-14, and metadata provided by the SEM. A correlation can be seen between the mean height and the mean diameter as a function of the radial position. This observation aligns with the earlier statements about the influence of the pre-shaped SiNC initial top diameter on the morphology after thermal oxidation. Smaller initial diameters lead to smaller self-limited diameters. Assuming that a 30 ± 5 nm nanowire self-limits to 5 nm. A bottled nanowire with a smaller top diameter will meet the 30 ± 5 criterium somewhere further down from the top. This will result in a reduced height after oxidation. For higher temperatures, the final diameter will be smaller and thus will yield an even smaller final height. This is exactly what is observed in Fig. 8a and b. Furthermore, height measurements taken on SEM images of coated SiNC and stripped SiNC arrays, as shown in Additional file 1: Figs. SI-10 and SI-11, show a similar trend. Additional file 1: Fig. SI-12 shows the height of the stripped and coated arrays. Measurements of the height of the coated arrays show height variations with a standard deviation of ± 5 nm (1% relative deviation) within a single SEM image for all oxidation temperatures. This height variation may likely be attributed to the error caused by manual measurement of the height in a cross-sectional SEM image, since the scale is 1.4 nm/pixel. Additional file 1: Fig. SI-12 also shows the calculated difference in heights between coated and stripped SiNC arrays, for different temperatures and substrate positions. The difference is more prevalent for increased oxidation temperatures and at positions further away from the center of the substrate.

### Influence of the Mask Pattern

To investigate the cause of the radial height distribution, a series of top-view SEM images were taken after different steps in the fabrication procedure. An automated MATLAB script was used to determine the radial diameter of the top of the pre-shaped SiNCs, SiNWs, and the BARC columnar mask, used for the fabrication of the SiNW array [51, 52]. This script was performed at different locations on the substrate surface and the radial coordinates were again determined using the metadata provided by the SEM system. In this case, extracting ${r}_{c}$ is easier, because measurements are taken on intact (i.e., non-cleaved) substrates. The top diameter variation of the pre-shaped SiNCs, as plotted in Fig. 8b, was also present after CM-ICP RIE of the silicon nanowires before rat-IBE pre-shaping. In fact, this variation was also found for measurements conducted on the BARC polymer columns before CM-ICP RIE of the SiNWs, as shown in Additional file 1: Fig. SI-14. Additional file 1: Fig. SI-14 contains examples of the circle fitting algorithm, histogram plots of the top diameter distributions, and interval plots of the measured top diameter as a function of ${r}_{\mathrm{c}}$. The results indicate that the radial diameter variations of the structures are defined during the RIE etching of the BARC polymer in a nitrogen plasma and progresses through the fabrication process. From the literature, it is known that the BARC nanocolumns etch isotropically in N_2_ plasma at a low rate [51]. Etching characterization of the radial thickness variation on a wafer-scale planar BARC film by RIE in an *N*_2_ plasma was conducted by ellipsometry. Measurements on the radial distribution of the planar BARC film thickness before RIE is applied are added in Additional file 1: Fig. SI-15 and show no radial thickness variation. After etching the planar film by RIE in *N*_2_ a radial thickness variation was revealed where the etch rate increases radially outward. The BARC is etched fastest on the outer positions, as shown in Additional file 1: Fig. SI-16. This will yield BARC columns with smaller diameters at the edge of the substrate as measured in Additional file 1: Fig. SI-14 and explains the radial top diameter variation observed in the pre-shaped SiNCs.

We finalize by stating that the combination of CM-ICP RIE of silicon, IBE step, and thermal oxidation provide good control at the wafer scale. The fabrication method follows the initial mask design which, in this case, is a BARC column polymer mask with some radial diameter variation. Considering that different SiNW arrays were presented, we suggest that rat-IBE combined with thermal oxidation can be applied to different nanowire structures for sharpening purposes. We also would like to mention that numerical modeling of the 3D growth of the t-SiO_2_ may answer the question of whether there is a significant influence of the internal angle on the film growth, where recent models considering fully coupled chemo-mechanical interaction might prove beneficial [67, 74–76]. A correct model may facilitate the choice of a temperature where oxidation self-limits at the desired diameter, taking the initial pre-shaped SiNC top diameter as an input. Such modeling work is considered outside the scope of this paper and will be a topic of future work.

## Conclusion

Using a combination of additive hybrid lithography, continuous mode reactive ion etching, rotated angled and timed ion beam etching, self-limited thermal oxidation and wet chemical etching of the grown silicon dioxide provide a way to obtain high-density, high aspect ratio, periodic and vertically aligned sharp single-crystalline silicon nanocones derived from a silicon nanowire structure. The silicon nanocones were organized in a square periodic lattice with 250 nm pitch, giving 1.6 billion structures per square centimeter. The nanocones were obtained in several mm^2^-sized windows located across a 100-mm-diameter single-crystalline silicon (100) substrate. Tip radii of curvature < 3 nm were measured. The structures were vertically aligned, showing a height variation < 5 nm (< 1%) for seven adjacent nanocones, and a height inhomogeneity < 80 nm (< 16%) across the wafer scale. This inhomogeneity was explained by inhomogeneity present in the initial polymer BARC mask used for continuous mode reactive ion etching of the silicon nanowires. Thus, the method shows good transferability, following the initial mask design.

## Supplementary Information

**Additional file 1: Fig. SI-1.** Cross-sectional SEM image of an input SiNW array used for characterization of CM-ICP RIE and rat-IBE etching in Figs. SI-2 and SI-3. The scale bar represents 500 nm. **Fig. SI-2.** SEM images on a cleaved cross section of the SiNW array after ICP RIE of the SiNWs in an argon plasma as a function of etching times (1, 5, 10, and 20 min). The power applied to the ICP generator was 800 W, the argon flow into the plasma chamber was set at 20 sccm, the pressure was kept at 18 mTorr, the applied power to the CCP RF generator was 108 W yielding a DC potential of 200 V between the plasma cloud and the wafer chuck. The wafer chuck temperature was set at 0 °C. The images show a start of tapering on the SiNW between 1 and 5 min, which stabilizes in time. An extended etching time shows a decrease in length of the SiNWs, while the wires also get an increased diameter. This is similar to the observations made by Hung et al. [21] The image on the far-right shows a cross-sectional view, revealing that the obtained tapered SiNWs contain a core material that is different from its apparent shell. It is hypothesized that the core is the crystalline SiNW with an amorphous silicon shell. The shell consists of silicon that redeposited during the etching cycle. **Fig. SI-3.** Micrograph of a cross section obtained by SEM after step b in Fig. 1 of the main text. The results show the SiNCs typically obtained by applying the additive hybrid lithography mask method, RIE in a mixed-mode SF_6_+C_4_F_8_ etching step, and an IBE step of 10:00 (mm:ss) at different incident angles. Upon an increase in the ion beam incident angle, the lateral etch rate increases, up to the point where the SiNWs are constricted in the lateral direction forming sharp tips. However, too large incident angles create additional surface roughness at the flat silicon surface. Lastly, the shadowing effect of the neighboring structures can be observed through a change of the taper angle of the SiNC at different height locations along the cylindrical symmetry axis. **Fig. SI-4.** Micrographs of a cross section obtained by SEM after step a, no IBE, or b, other pictures, in Fig. 1 of the main text. For these samples the etching time was reduced to 5:00 (mm:ss), to prevent lateral constriction and to facilitate extraction of the SiNW dimensions after IBE. **Fig. SI-5.** Cross-sectional SEM images of the pre-shaped SiNC array used for characterization of the thermal oxidative sharpening process. The scale bar on left represents 1000 nm. The scale bar on the right represents 200 nm. **Fig. SI-6.** A single substrate was divided in four quarters and subjected to dry thermal oxidation at 850 °C. Substrates were removed from the furnace at 30-minute intervals between a total oxidation time of 01:50 (hh:mm) and 03:20 (hh:mm). An ellipsometric line scan was performed to measure the grown SiO_2_ thickness on the flat surface surrounding the individual samples. Additionally, SEM images at the cross section of the SiNC arrays were recorded before and after stripping the SiO_2_ to visualize the SiNC shape. Measurements were taken at the (2,2), (2,3) (3,2), and (3,3) locations (see Fig. 7 in the main text). Although the layer thickness seems to still increase on the flat substrate for extended oxidation times as extracted from the ellipsometry measurements, the vertical alignment of the SiNC appears unaltered, indicating a stress mediated self-limiting effect. The scale bars represent 100 nm. **Fig. SI-7.** The micrograph depicted on the left was obtained through SEM and shows the SiNC array achieved by oxidation of a pre-shaped SiNC at 950 °C and stripping of the deposited Si_3_N_4_ and t-SiO_2_. The encircled areas show defects of the SiNC due to oxidation. The TEM image on the right shows that a silicon tip is enclosed inside a silicon dioxide layer. The Si_3_N_4_ is deposited conformally on top of the oxide, thus the defect is a result of oxidation. **Fig. SI-8.** Micrographs obtained by scanning electron microscopy, collected for different oxidation temperatures after removal of the grown oxide. The scale bars represent 100 nm. **Fig. SI-9.** A photograph of a 100-mm-diameter (001)-oriented silicon substrate containing SiNC arrays at different locations of the wafer. The pattern was obtained through the additive hybrid lithography method. **Fig. SI-10.** Micrographs of a cross section of the coated, left column, and stripped, right column, SiNC array obtained by SEM after step d, coated, and e. Stripped, as shown in Fig.1 of the main text. The images were recorded at the discrete substrate position (1,1) for different oxidation temperatures. The scale bar represents 200 nm. **Fig. SI-11.** Micrographs of a cross section of the coated, left column, and stripped, right column, SiNC array obtained by SEM after step d, coated, and e. Stripped, as shown in Fig.1 of the main text. The images were recorded at the discrete substrate position (2,2) for different oxidation temperatures. The scale bar represents 200 nm. **Fig. SI-12.** Result from height measurements were taken on cross-sectional SEM images for different oxidation temperatures as a function of the substrate location where the measurement was performed. Measurements were taken on images like those displayed in Figs. SI-10 and SI-11. The data are presented as interval plots for the height of the coated SiNC, the stripped SiNC and the difference between the coated and stripped array. A 95% CI is shown for *N* =14 individual cones. **Fig. SI-13.** Interval plot of the SiNC heights for three oxidation temperatures measured at different window locations (row, column) on the wafer, after stripping the Si_3_N_4_ and SiO_2_ layers. The edge and center measurements in a single window location are pooled into the same interval plot. Fig. 7 in the main text can be used as a reference for the locations of the windows. A 95% CI is given with *N* =14 stripped cone height measurements for individual interval plots. It is observed that the height distribution has a parabolic shape as a function of the row position indicator. This could be due to misalignment of the mask pattern origin with respect to the substrate origin. **Fig. SI-14.** Representation of the top diameter extraction measurement. First, a top-view SEM image is made, after which it is binarized. A circle fitting algorithm in MATLAB is used to detect and fit the individual tops BARC columns, SiNWs and SiNCs. Together with the metadata provided by the SEM system, the measured pixel diameter is converted to a metric scale and the mean and standard deviations of the top diameters are calculated. A histogram plot shows the near normal distribution of the collected diameters for individual window locations on the substrate. Lastly, an interval plot is used to show the progression of the top diameters as a function of the radial location calculated as explained in the main text. The interval plots show a 95% CI with *N* > 50 for individual intervals. **Fig. SI-15.** Ellipsometry measurements on a planar BARC film for different substrates prior to etching characterization by RIE in N2. The images show a uniform thickness profile, indicating the absence of a radial thickness distribution. The substrate maps are obtained through a linear interpolation between the 13 measured points. **Fig. SI-16.** The motivation for these experiments was to characterize wafer-scale variation of N_2_ dry etching step in a CCP RIE. Different time intervals were applied because there was a hypothesis that temperature might be influential. For example, at first 5216 was etched for 2 min, then 5215 was etched for 4 min, followed by etching of 5216 for an additional 2 min. Both substrates have spent 4 min in the plasma, where the thermal build-up for 5215 might be increased because of the uninterrupted etching. The results show thickness maps of the BARC thickness after etching at said time intervals obtained by ellipsometry. A clear radial thickness variation is observed. Moreover, a difference was observed between interrupted and uninterrupted RIE, indicating that the etching of BARC in N_2_ plasma can be influenced by surface temperature.

## Data Availability

The datasets used and/or analyzed during the current study are available from the corresponding author upon reasonable request. More information regarding supporting results is added to the electronic supporting information. This file contains measurement results and descriptions referenced throughout the main text.

## Abbreviations

(C): Center of a SiNC array
(E): Edge of a SiNC array
(n): Not overetched during rat-IBE
(y): Overetched during rat-IBE
AR: Aspect ratio
CI: Confidence interval
CM-ICP RIE: Continuous mode inductively coupled plasma reactive ion etching
CM-RIE: Continuous mode reactive ion etching
c-Si: Single-crystalline silicon
DTL: Displacement Talbot lithography
FIB: Focused ion beam etching
H_2_O_2_: Hydrogen peroxide
H_3_PO_4_: Phosphoric acid
HCl: Hydrochloric acid
HF: Hydrofluoric acid
IBE: Ion beam etching
ICP RIE: Inductively coupled plasma reactive ion etching
LPCVD: Low-pressure chemical vapor deposition
oDTL: Orthogonal displacement Talbot lithography
Pt: Platinum
QDR: Quick-dump rinsing
rat-IBE: Rotated angled timed ion beam etching
RIE: Reactive ion etching
SEM: Scanning electron microscopy
Si_3_N_4_: Stoichiometric silicon nitride
SiNC: Silicon nanocone
SiNW: Silicon nanowire
SiO_2_: Silicon dioxide
TEM: Transmission electron microscopy
t-SiO_2_: Thermally grown silicon dioxide
