# Supplementary material for: Large Dense Periodic Arrays of Vertically Aligned Sharp Silicon Nanocones
Source: Nanoscale Res Lett. 2022 Oct 16;17:100. doi: 10.1186/s11671-022-03735-y (PMC9573847; doi:10.1186/s11671-022-03735-y)
Supplement: Supplementary file 1 — Additional file 1: Fig. SI-1. Cross-sectional SEM image of an input SiNW array used for characterization of CM-ICP RIE and rat-IBE etching in Figs. SI-2 and SI-3. The scale bar represents 500 nm. Fig. SI-2. SEM images on a cleaved cross section of the SiNW array after ICP RIE of the SiNWs in an argon plasma as a function of etching times (1, 5, 10, and 20 min). The power applied to the ICP generator was 800 W, the argon flow into the plasma chamber was set at 20 sccm, the pressure was kept at 18 mTorr, the applied power to the CCP RF generator was 108 W yielding a DC potential of 200 V between the plasma cloud and the wafer chuck. The wafer chuck temperature was set at 0 °C. The images show a start of tapering on the SiNW between 1 and 5 min, which stabilizes in time. An extended etching time shows a decrease in length of the SiNWs, while the wires also get an increased diameter. This is similar to the observations made by Hung et al. [21] The image on the far-right shows a cross-sectional view, revealing that the obtained tapered SiNWs contain a core material that is different from its apparent shell. It is hypothesized that the core is the crystalline SiNW with an amorphous silicon shell. The shell consists of silicon that redeposited during the etching cycle. Fig. SI-3. Micrograph of a cross section obtained by SEM after step b in Fig. 1 of the main text. The results show the SiNCs typically obtained by applying the additive hybrid lithography mask method, RIE in a mixed-mode SF6+C4F8 etching step, and an IBE step of 10:00 (mm:ss) at different incident angles. Upon an increase in the ion beam incident angle, the lateral etch rate increases, up to the point where the SiNWs are constricted in the lateral direction forming sharp tips. However, too large incident angles create additional surface roughness at the flat silicon surface. Lastly, the shadowing effect of the neighboring structures can be observed through a change of the taper angle of the SiNC at different [file 11671_2022_3735_MOESM1_ESM.docx]

| **Electronic supporting information: Large dense periodic arrays of vertically aligned sharp silicon nanocones**  Dirk Jonker^1^(**🖂**), Erwin J.W. Berenschot^1^, Niels R. Tas^1^, Roald M. Tiggelaar^2^, Arie van Houselt^3^, Han J.G.E. Gardeniers^1^  *^1 Mesoscale Chemical Systems, MESA+ Institute, University of Twente, P.O. Box 217, 7500 AE Enschede, The Netherlands.^*  *^2 NanoLab cleanroom, MESA+ Institute, University of Twente, P.O. Box 217, 7500 AE, Enschede, The Netherlands^*  *^3 Physics of Interfaces and Nanomaterials, MESA+ Institute, University of Twente, P.O. Box 217, 7500 AE Enschede, The Netherlands.^* |
| --- |
|  |


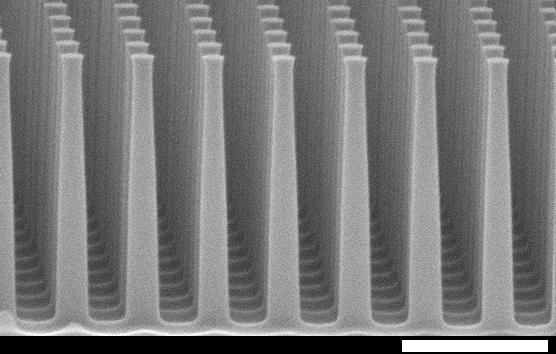


Figure SI-1. Cross-sectional SEM image of an input SiNW array used for characterization of CM-ICP RIE and rat-IBE etching in Fig SI-2 and SI-3. The scale bar represents 500 nm.


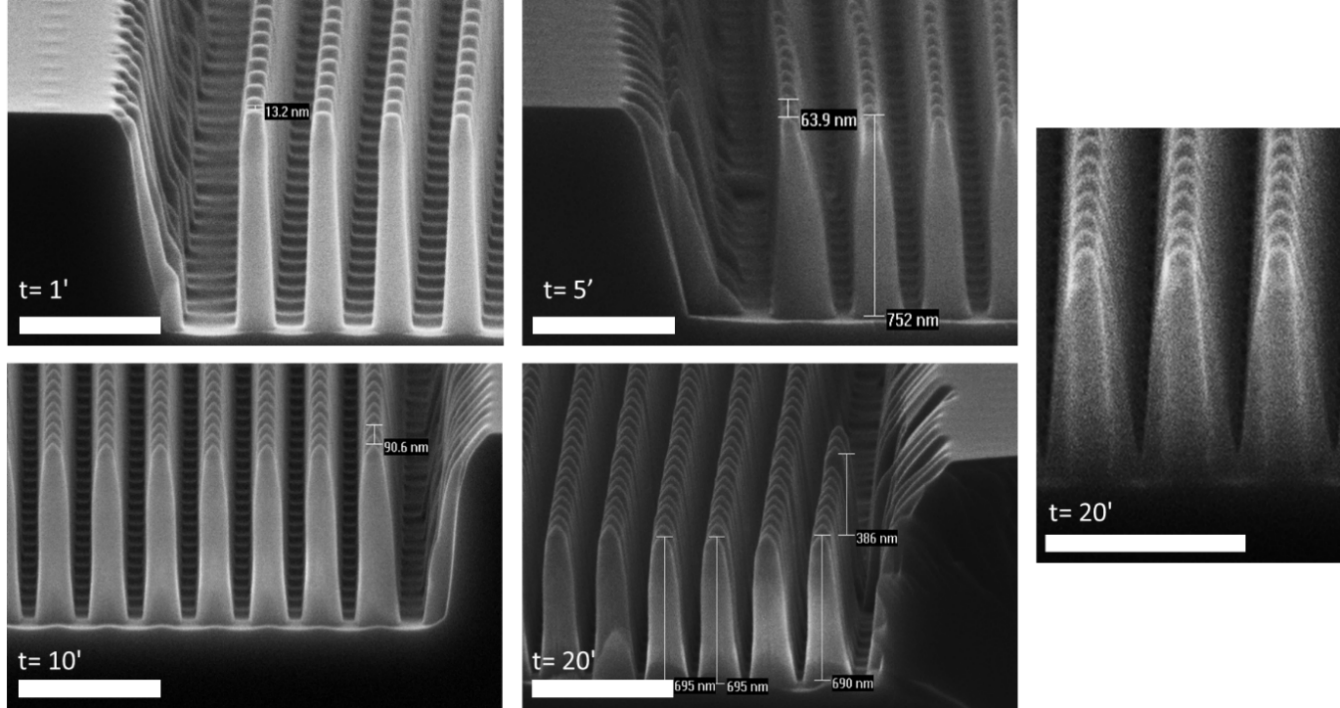


Inclination

Figure SI-2. SEM images on a cleaved cross-section of the SiNW array after ICP-RIE of the SiNWs in an argon plasma as a function of etching times (1, 5, 10, and 20 minutes). The power applied to the ICP generator was 800W, the argon flow into the plasma chamber was set at 20 sccm, the pressure was kept at 18 mTorr, the applied power to the CCP RF generator was 108W yielding a DC potential of 200V between the plasma cloud and the wafer chuck. The wafer chuck temperature was set at 0 °C. The images show a start of tapering on the SiNW between 1 and 5 minutes, which stabilizes in time. An extended etching time shows a decrease in length of the SiNWs, while the wires also get an increased diameter. This is similar to the observations made by Hung et al.[21] The image on the far-right shows a cross-sectional view, revealing that the obtained tapered SiNWs contain a core material that is different from its apparent shell. It is hypothesized that the core is the crystalline SiNW with an amorphous silicon shell. The shell consists of silicon that redeposited during the etching cycle.


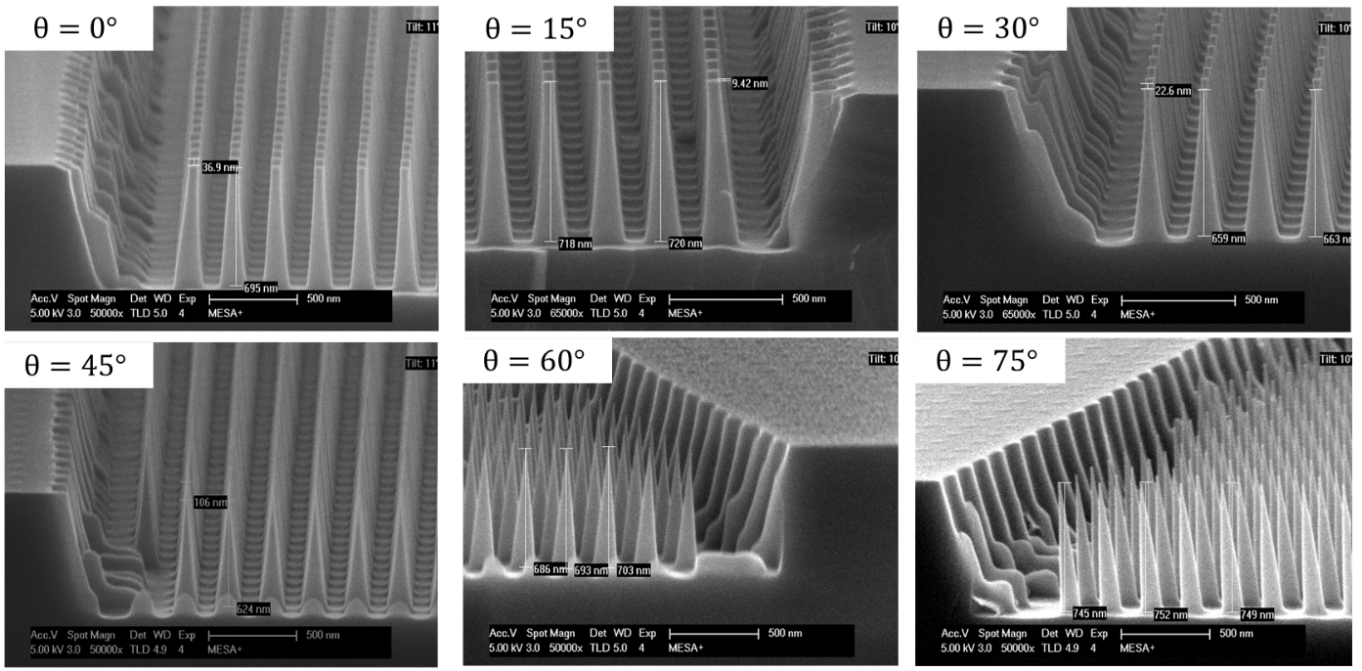


Figure SI-3. Micrograph of a cross-section obtained by SEM after step b) in Fig. 1 of the main text. The results show the SiNCs typically obtained by applying the additive hybrid lithography mask method, RIE in a mixed-mode SF_6_+C_4_F_8_ etching step, and an IBE step of 10:00 (mm:ss) at different incident angles. Upon increase of the ion beam incident angle, the lateral etch-rate increases, up to the point where the SiNWs are constricted in the lateral direction forming sharp tips. However, too large incident angles create additional surface roughness at the flat silicon surface. Lastly, the shadowing effect of the neighbouring structures can be observed through a change of the taper angle of the SiNC at different height locations along the cylindrical symmetry axis.


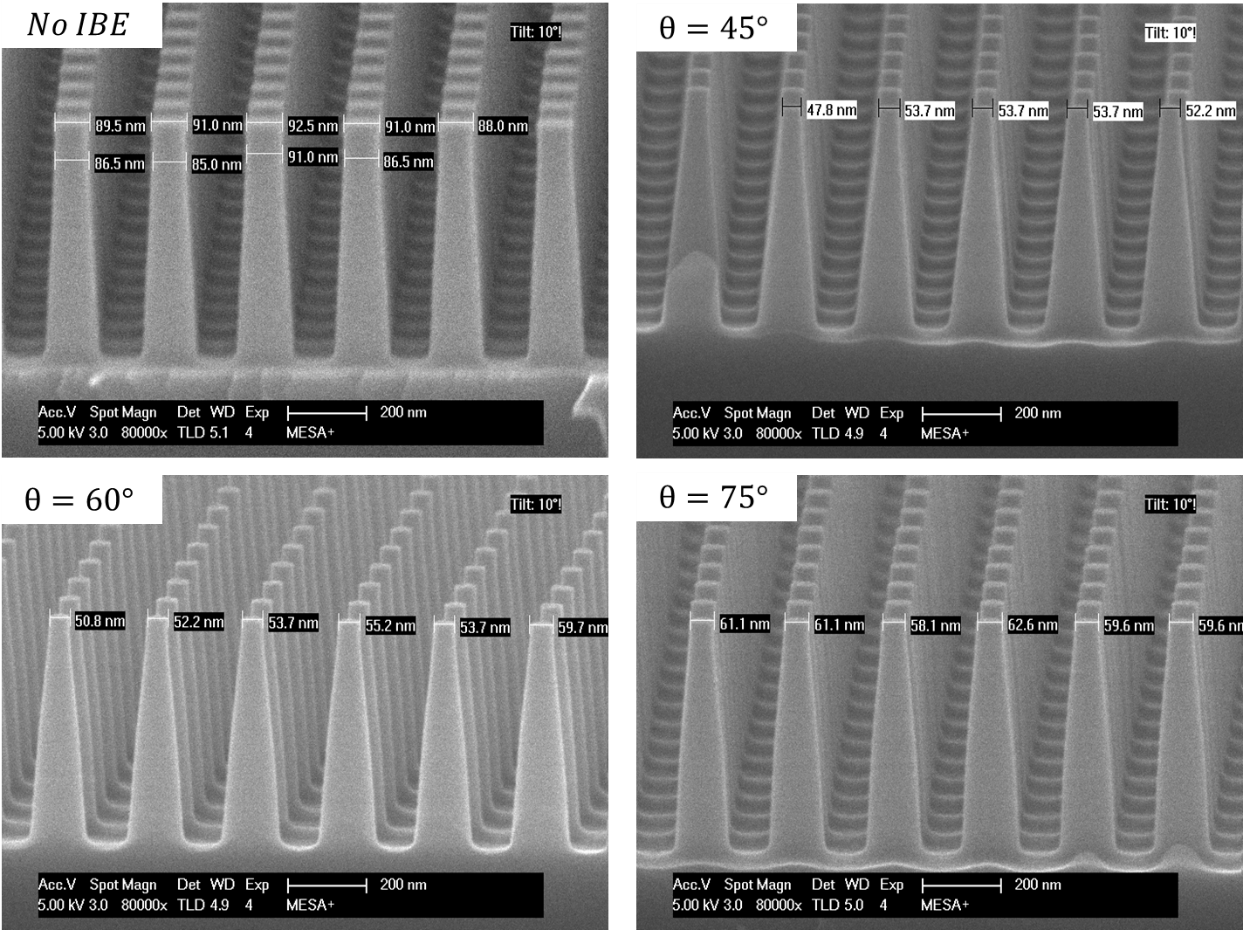


Figure SI-4. Micrographs of a cross-section obtained by SEM after step a), no IBE, or b), other pictures, in Fig. 1 of the main text. For these samples the etching time was reduced to 5:00 (mm:ss), to prevent lateral constriction and to facilitate extraction of the SiNW dimensions after IBE.


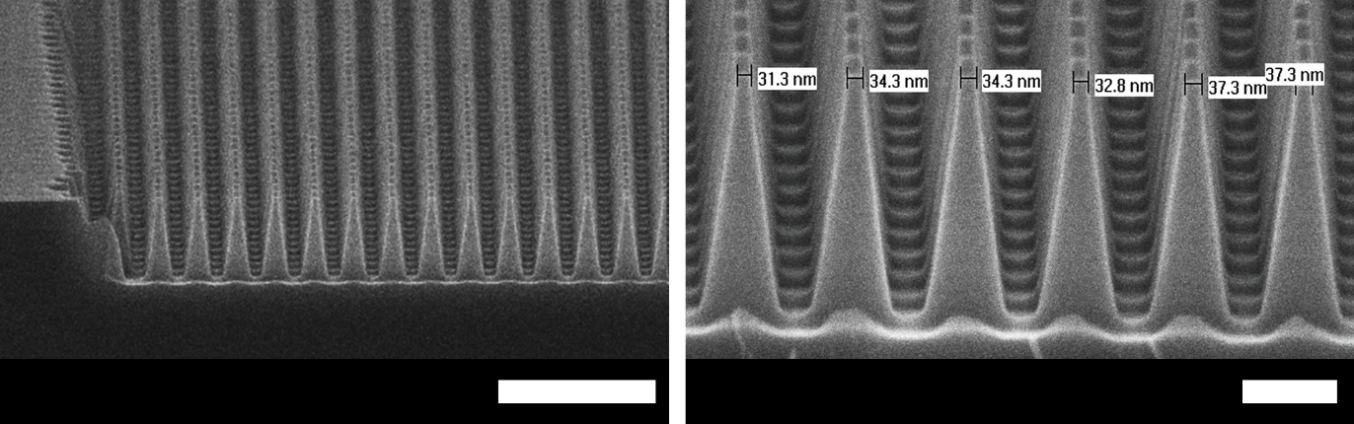


Figure SI-5. Cross-sectional SEM images of the pre-shaped SiNC array used for characterization of the thermal oxidative sharpening process. The scale bar on left represents 1000 nm. The scale bar on the right represents 200 nm.


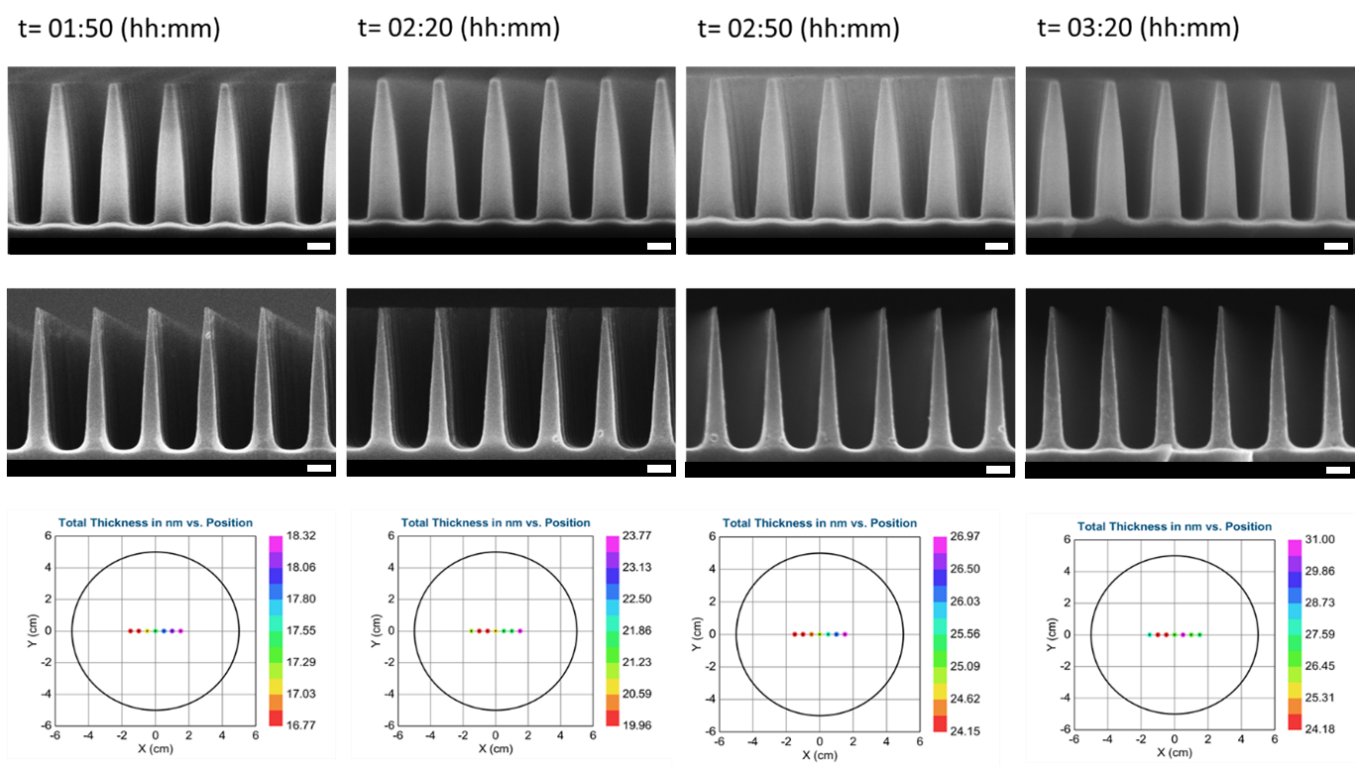


Figure SI-6. A single substrate was divided in four quarters and subjected to dry thermal oxidation at 850 °C. Substrates were removed from the furnace at 30-minute intervals between a total oxidation time of 01:50 (hh:mm) and 03:20 (hh:mm). An ellipsometric line scan was performed to measure the grown SiO_2_ thickness on the flat surface surrounding the individual samples. Additionally, SEM images at the cross-section of the SiNC arrays were recorded before and after stripping the SiO_2_ to visualize the SiNC shape. Measurements were taken at the (2,2), (2,3) (3,2) and (3,3) locations (see Fig. 7 in the main text). Although the layer thickness seems to still increase on the flat substrate for extended oxidation times as extracted from the ellipsometry measurements, the vertical alignment of the SiNC appears unaltered, indicating a stress mediated self-limiting effect. The scale bars represent 100 nm.


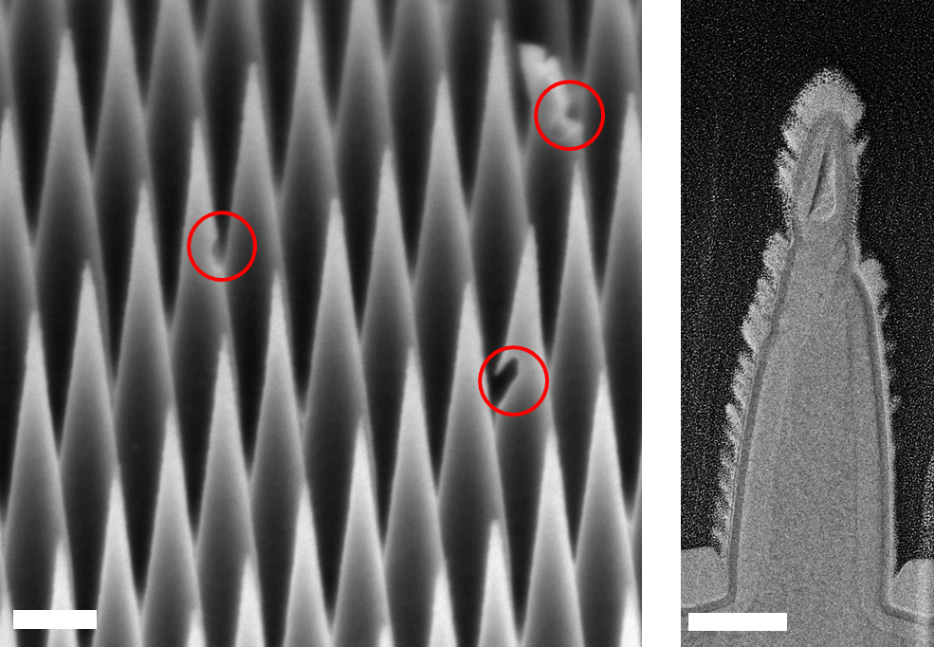


Figure SI-7. The micrograph depicted on the left was obtained through SEM and shows the SiNC array achieved by oxidation of a pre-shaped SiNC at 950 °C and stripping of the deposited Si_3_N_4_ and t-SiO_2_. The encircled areas show defects of the SiNC due to oxidation. The TEM image on the right shows that a silicon tip is enclosed inside a silicon dioxide layer. The Si_3_N_4_ is deposited conformally on top of the oxide, thus the defect is a result of oxidation.


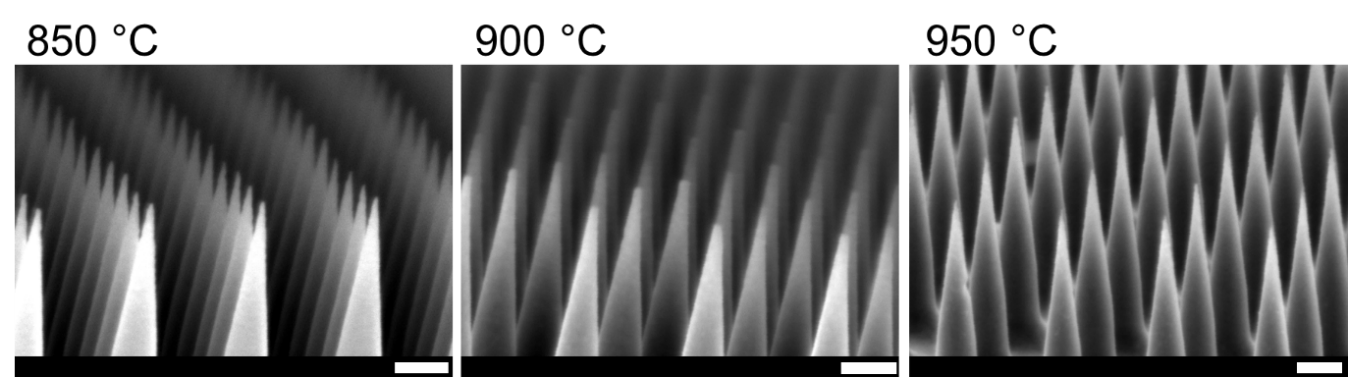


Figure SI-8. Microcraphs obtained by scanning electron microscopy, collected for different oxidation temperatures after removal of the grown oxide. The scale bars represent 100 nm.


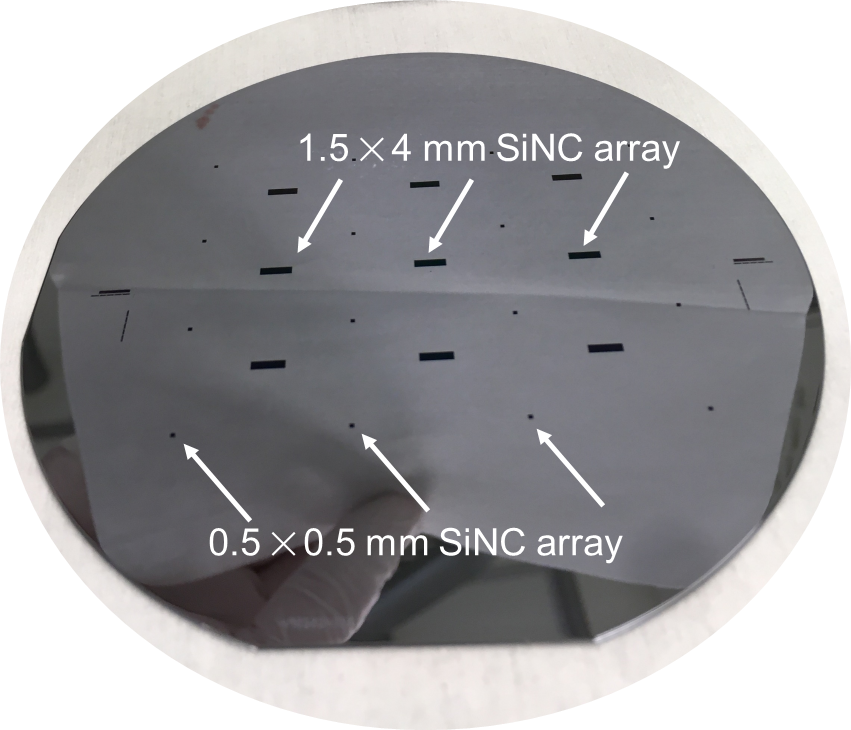


Figure SI-9. A photograph of a 100 mm diameter (001)-oriented silicon substrate containing SiNC arrays at different locations of the wafer. The pattern was obtained through the additive hybrid lithography method.


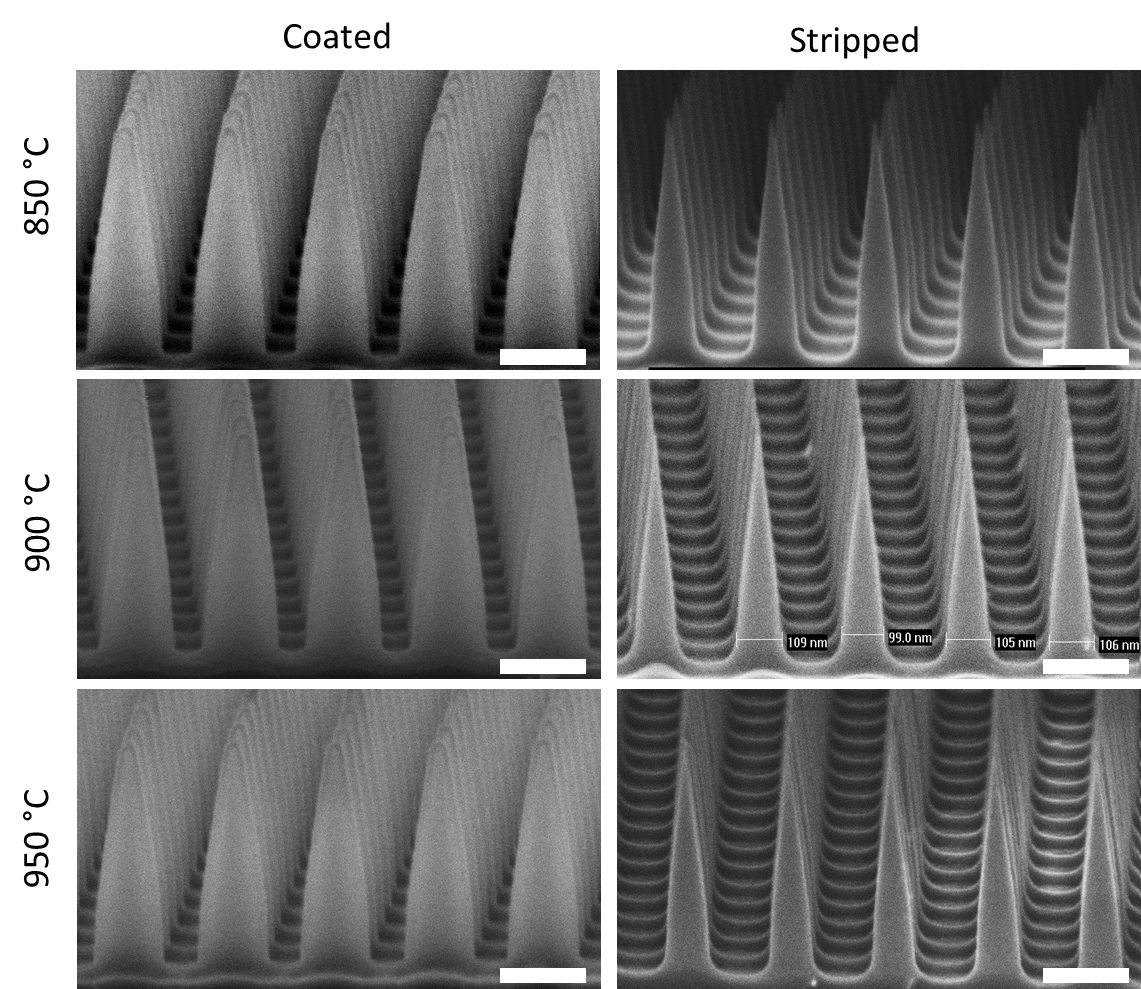


Figure SI-10. Micrographs of a cross-section of the coated, left column, and stripped, right column, SiNC array obtained by SEM after step d), coated, and e). stripped, as shown in Fig .1 of the main text. The images were recorded at the discrete substrate position (1,1) for different oxidation temperatures. The scale bar represents 200 nm.


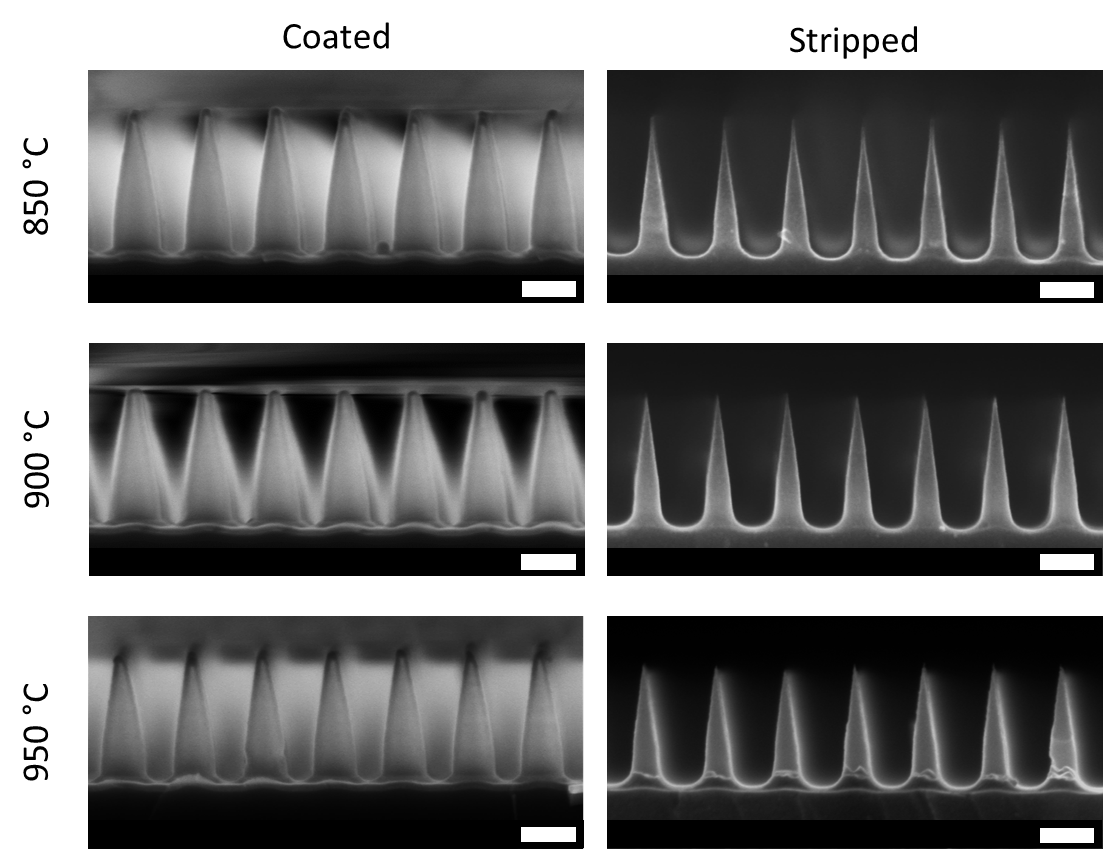


Figure SI-11. Micrographs of a cross-section of the coated, left column, and stripped, right column, SiNC array obtained by SEM after step d), coated, and e). stripped, as shown in Fig .1 of the main text. The images were recorded at the discrete substrate position (2,2) for different oxidation temperatures. The scale bar represents 200 nm.


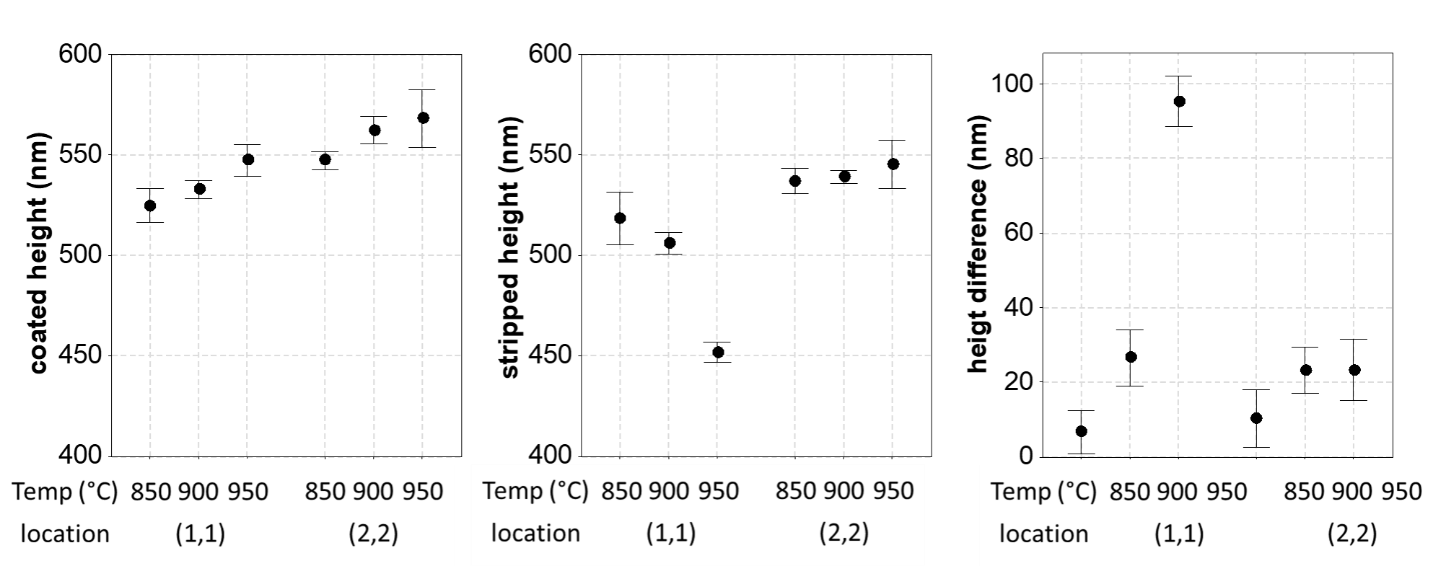


Figure SI-12. Result from height measurements performed on cross-sectional SEM images for different oxidation temperatures as a function of the substrate location where the measurement was performed. Measurements were conducted on images like those displayed in Fig SI-10 and Fig SI-11. The data is presented as interval plots for the height of the coated SiNC, the stripped SiNC and the difference between the coated and stripped array. A 95% CI is shown for N =14 individual cones.


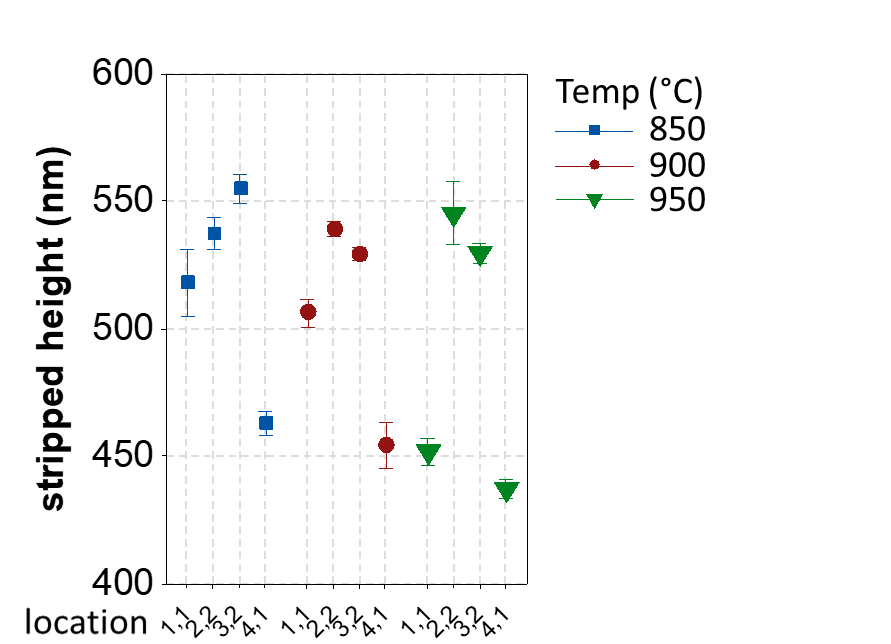


Figure SI-13 Interval plot of the SiNC heights for three oxidation temperatures measured at different window locations (row, column) on the wafer, after stripping the Si_3_N_4_ and SiO_2_ layers. The edge and center measurements in a single window location are pooled into the same interval plot. Fig. 7 in the main text can be used as a reference for the locations of the windows. A 95% CI is given with N =14 stripped cone height measurements for individual interval plots. It is observed that the height distribution has a parabolic shape as a function of the row position indicator. This could be due to misalignment of the mask pattern origin with respect to the substrate origin.


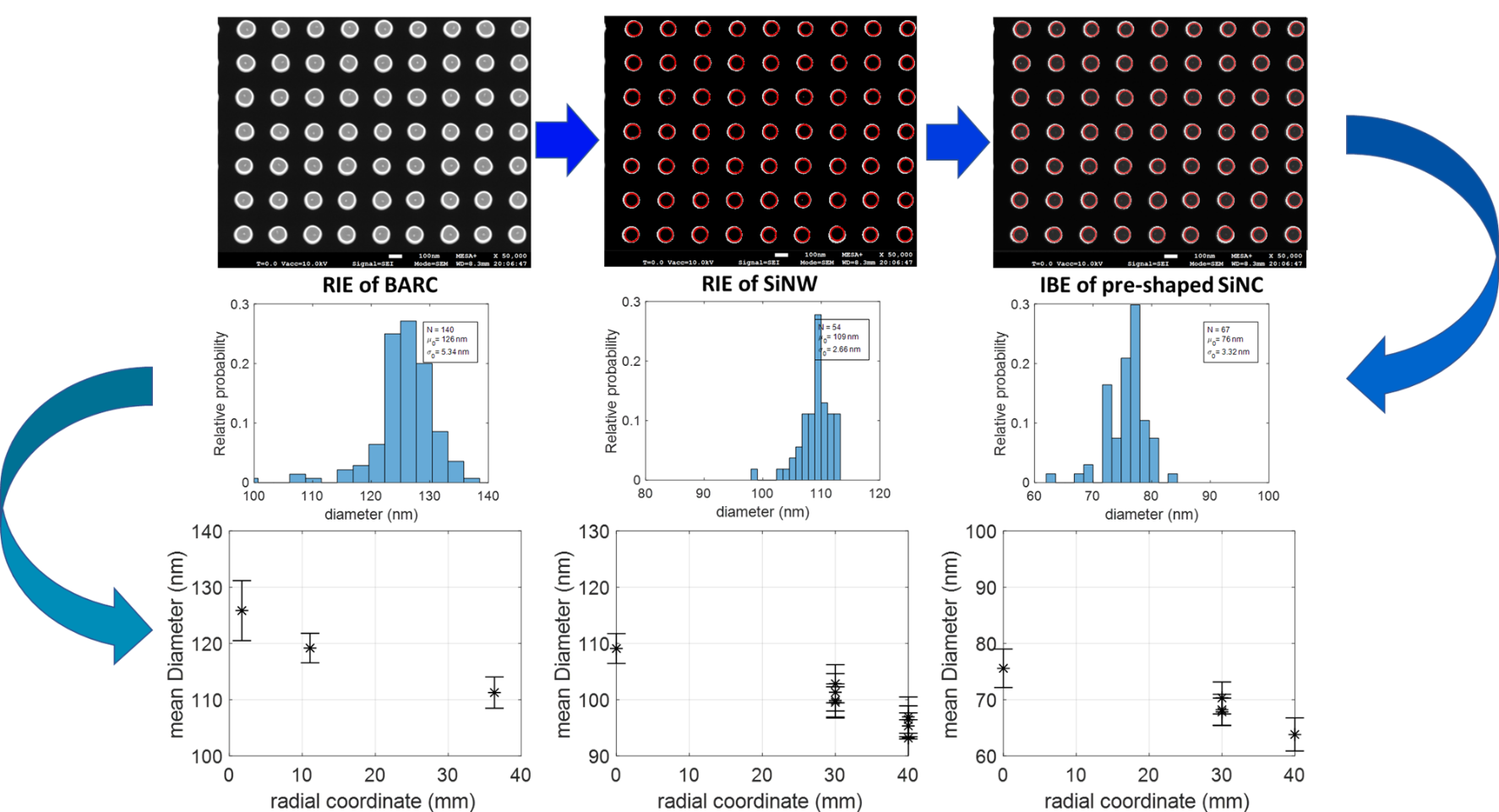


Figure SI-14. Representation of the top diameter extraction measurement. First, a top-view SEM image is made, after which it is binarized. A circle fitting algorithm in MATLAB is used to detect and fit the individual tops BARC columns, SiNWs and SiNCs. Together with the metadata provided by the SEM system, the measured pixel diameter is converted to a metric scale and the mean and standard deviations of the top diameters are calculated. A histogram plot shows the near normal distribution of the collected diameters for individual window locations on the substrate. Lastly, an interval plot is used to show the progression of the top diameters as a function of the radial location calculated as explained in the main text. The interval plots show a 95% CI with N>50 for individual intervals.


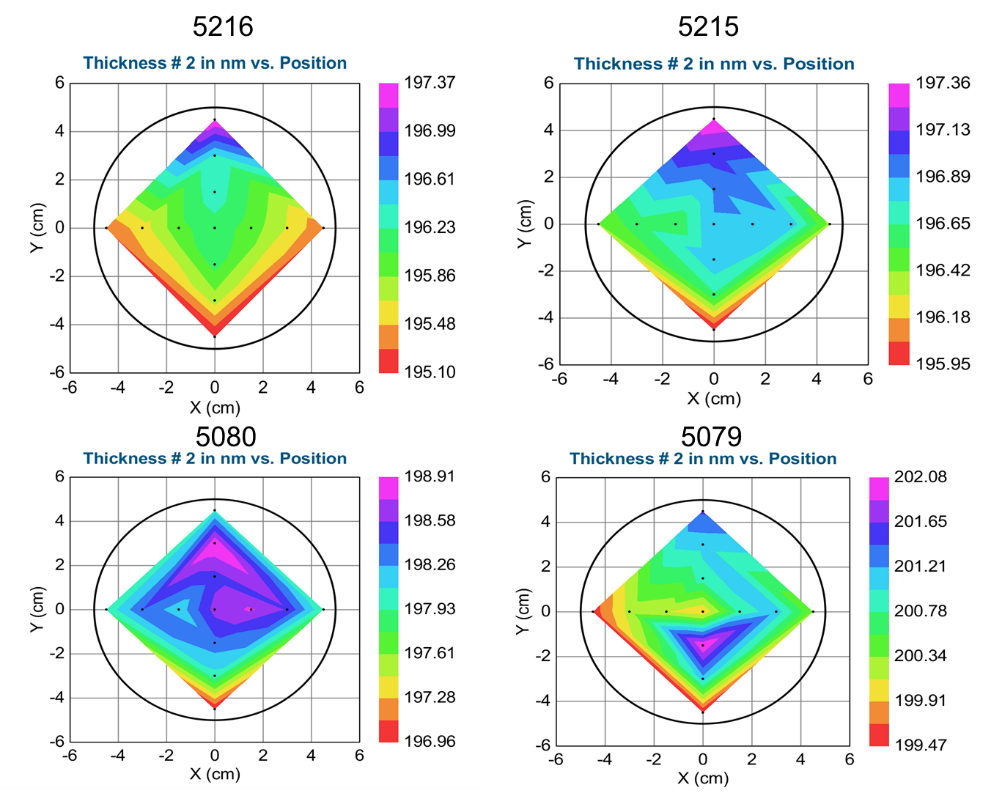


Figure SI-15. Ellipsometry measurements on a planar BARC film for different substrates prior to etching characterization by RIE in N_2_. The images show a uniform thickness profile, indicating the absence of a radial thickness distribution. The substrate maps are obtained through a linear interpolation between the 13 measured points.


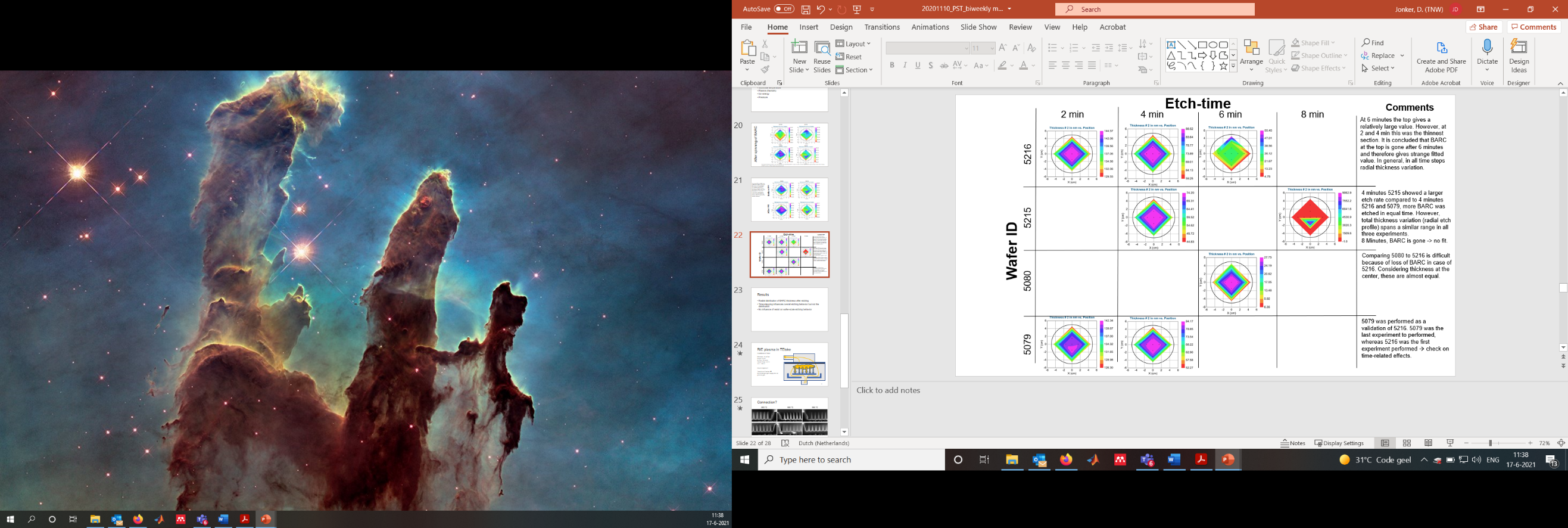


Figure SI-16. The motivation for these experiments was to characterize wafer-scale variation of N_2_ dry etching step in a CCP RIE. Different time intervals were applied because there was a hypothesis that temperature might be influential. For example, at first 5216 was etched for 2 minutes, then 5215 was etched for 4 minutes, followed by etching of 5216 for an additional 2 minutes. Both substrates have spent 4 minutes in the plasma, where the thermal build-up for 5215 might be increased because of the uninterrupted etching. The results show thickness maps of the BARC thickness after etching at said time intervals obtained by ellipsometry. A clear radial thickness variation is observed. Moreover, a difference was observed between interrupted and uninterrupted RIE, indicating that the etching of BARC in N_2_ plasma can be influenced by surface temperature.
